# Quantitative proteomics reveals *Polygonum perfoliatum L.* ameliorates hepatic steatosis by promoting PPARs/CPT1A/CPT2-mediated fatty acid β-oxidation

**DOI:** 10.3389/fphar.2023.1016129

**Published:** 2023-03-23

**Authors:** Guanjie Liu, Ling Chang, Yihan Qian, Jiacheng Lin, Zhi Shang, Min Xu, Fang Wang, Xuehua Sun, Yun Jiang, Yueqiu Gao, Xiaoni Kong

**Affiliations:** ^1^ Central Laboratory, Department of Liver Diseases, ShuGuang Hospital Affiliated to Shanghai University of Chinese Traditional Medicine, Shanghai, China; ^2^ Department of Gastroenterology, The Seventh People’s Hospital of Shanghai University of Traditional Chinese Medicine, Shanghai, China; ^3^ Department of Liver Diseases, Shuguang Hospital Affiliated to Shanghai University of Traditional Chinese Medicine, Shanghai, China

**Keywords:** quantitative proteomics, hepatic steatosis, polygonum perfoliatum L., fatty acid β oxidation, lipid metabolism

## Abstract

Non-alcoholic fatty liver disease (NAFLD) is a predominant contributor to end-stage liver disease in the forthcoming decades. *Polygonum perfoliatum L.* (PPL) is an herbal medicine with anti-lipid peroxidation and anti-inflammatory properties. However, detailed hepatoprotective effects of PPL against NAFLD and its underlying mechanisms are not fully understood. Here, we found that PPL protects against high fat diet (HFD)-induced hepatic steatosis, lipid peroxidation, and glucose-lipid metabolism dysfunction in NAFLD mice. We therefore performed a label-free quantitative proteomic profiling analysis to determine the effect of PPL treatment on liver tissue proteomics and identified that activated PPARs/CPT1A/CPT2-mediated hepatic fatty acid β-oxidation (FAO) process was significantly altered. *In vitro* treatment of hepatocytes with PPL confirmed this altered process and FAO inhibitor etomoxir (ETO) attenuated the lipid-lowering activity of PPL in hepatocytes. Ultra-high-performance liquid chromatography/Q Exactive-HFX (UPLC/QE-HFX) was used to determine the material basis of anti-NAFLD activity of PPL. Our results have demonstrated the efficacy and potential mechanisms of PPL as an effective pharmacological therapy of NAFLD.

## 1 Introduction

NAFLD is currently one of the most prevalent chronic liver diseases, accounting for approximately a quarter of the global epidemic, and its prevalence will continue to increase in the foreseeable future (Huang et al., 2021). NAFLD has been identified as a progressive disease that can lead to a series of complications, including hepatic fibrosis, non-alcoholic steatohepatitis, hepatocirrhosis, as well as hepatocellular carcinoma (Tsuchida et al., 2018; Anstee et al., 2019). NAFLD is not only associated with single-target lesions, but also commonly accompanied by systemic metabolic dysfunctions, such as hypertension, hyperlipidemia, hyperglycemia, adipose tissue accumulation, and inflammation (Milic et al., 2014; Kasper et al., 2021). Due to the increasing global burden of NAFLD and the unclear pathogenesis of NAFLD, development of novel and effective drugs for NAFLD pharmacological treatment remains a major challenge (Sodum et al., 2021).

FAO is an aerobic biological process and a major pathway for fatty acid degradation (Houten et al., 2016). Accordingly, FAO deficiency is the underlying pathological mechanism that leads to impaired lipid metabolism, which ultimately induces metabolic syndrome (Merritt et al., 2018). During FAO process, fatty acid is converted to acyl-coenzyme A (acyl-CoA) by acyl-CoA synthase outside the mitochondria, which is subsequently transported into the mitochondria *via* CPT1 and CPT2 located in both outer and inner mitochondrial membranes, respectively. In addition, acyl-CoA is converted to acetyl coenzyme A (acetyl-CoA) by multiple dehydrogenases, which undergoes repeated tricarboxylic acid (TCA) cycle and ultimately generate large amounts of energy (Knottnerus et al., 2018; Adeva-Andany et al., 2019). Both CPT1 and CPT2 are key regulatory enzymes in the regulation of FAO, therefore, enhancing the activity of this pair of isozymes to facilitate fatty acids degradation would be one of the most promising directions for anti-NAFLD drug research and development.

According to traditional Chinese medicine (TCM), phlegm and dampness retention are the main causes of hyperlipidemia and NAFLD. PPL belongs to the Chinese herbal medicine, which has the effects of clearing heat and detoxification, dispelling phlegm, and relieving dampness, and is widely distributed in many regions of China (Liu et al., 2020). Therefore, PPL is also used clinically by practitioners in Chinese Medicine formulae for the treatment of hyperlipidemia and fatty liver disease. Modern pharmacological studies have shown that PPL contains a variety of pharmacological activities such as anti-diabetes, anti-liver fibrosis, anti-alcohol liver, anti-tumor, antibiosis, and anti-virus (Huang et al., 2008; Cao et al., 2011; Xing et al., 2011; Zhang et al., 2013; Zhang et al., 2014; Wei et al., 2016; Li et al., 2019). In this study, we found that PPL is rich in flavonoids, quinones, phenols, phenylpropanoids, terpenoids and other compounds, many of which have been shown to significantly ameliorate NAFLD pathology. Nevertheless, retrieval results indicated that no relevant data on the protective effects of PPL against NAFLD have been revealed.

In this study, we demonstrated the therapeutic effects of PPL on NAFLD progression based on an HFD-induced NAFLD model and characterized the protein expression profile, signaling pathway activation and histopathological changes. Our findings indicated that PPL might be a promising therapeutic agent for NAFLD through activation of FAO.

## 2 Materials and methods

### 2.1 Chemicals and reagents

PPL was purchased from Shuguang Hospital Affiliated to Shanghai University of Traditional Chinese Medicine (Shanghai, China). Voucher samples were deposited in the Central Laboratory of Shuguang Hospital. Serum biochemical indexes were measured using a full-automatic biochemical analyzer. MDA (Elabscience, E-BC-K025-M, China) and SOD (Elabscience, E-BC-K022-M, China) kits were purchased from Elabscience (Wuhan, China). Liver TC (APPLYGEN, E1026-105, China) and TG (APPLYGEN, E1013-50, China) levels were determined using APPLYGEN assay kits (Beijing, China). Primary antibodies included rabbit anti-PPARγ (CST, #2435, United States of America), rabbit anti-PPARα (Abclonal, A6697, China), mouse anti-CPT1A (Abcam, ab128568, United Kingdom), rabbit anti-CPT2 (Abcam, 181,114, United Kingdom), rabbit anti-SREBP2 (Abcam, ab30682, United Kingdom), mouse anti-HMGCR (Abcam, CL0260, United Kingdom), rabbit anti-CYP7A1 (Abcam, ab234982, United Kingdom), and actin (ABGENT, AP14779b, United States of America).

### 2.2 Preparation of PPL extract

To obtain an extract of PPL, 200 g PPL was soaked in 2 L distilled water at room temperature (RT) for 1 h and then heat to reflux for another 1 h. The extract was filtered, and the residue was subjected to a secondary extraction. The extracted mixture was concentrated under reduced pressure and further lyophilized to a fine powder to obtain 20.52 g lyophilized powder with a final yield of 10.26%.

### 2.3 Preparation of PPL metabolites from PPL treated mice serum

Male C57BL/6 mice were purchased from Jiesijie Research Institute (Shanghai, China) and divided into two groups. Mice in Group 1 were orally administered with PPL extract (400 mg/kg, equivalent to the clinical adult dose), and blood was collected from the orbit 1 h after administration. Mice in Group 2 were given saline and the serum was collected as a control group. Both serum samples were inactivated by heating at 56°C for 30 min. The protein precipitation was removed by adding twice the volume of acetonitrile (Aladdin, A298777, China) and the supernatant was transferred into a rotary evaporator (Yarong, RE-3000A, China) for concentration. The pure water was re-dissolved and desalted by a LC-C18 solid phase extraction column (CNW, SBQE-CA0955, China), and transferred into a vacuum centrifugal concentrator (SCIEMTZ, SCIENTZ-ILS, China). The concentrate was weighed, filtered, and sterilized by re-dissolving in pure water and stored at −80 °C until subsequent experiments.

### 2.4 Label free-based proteomics research

Total proteins were extracted from mice liver tissues sampled from HFD and HFD + PPL groups (n = 3) with 300 µL lysis buffer supplemented with 1 mM Phenylmethanesulfonyl fluoride (PMSF). Samples were centrifuged at 15,000 g for 15 min to remove insoluble particles, and then the precipitate was again excluded. After adding 5 times the volume of cold acetone and precipitating overnight at −20°C, the precipitate was collected by centrifugation at 12,000 g for 15 min at 4 °C. The precipitation was dried at RT for 3 min and then dissolved in SDS-lysis buffer for 2 h. Sample supernatant was collected and centrifuged again to remove the precipitate. The protein concentration was determined according to BCA method and samples were aliquoted to be stored at −80 °C. Base on BCA assay results, 100 μg of protein was extracted for filter-aided sample preparation (FASP) digestion. IAA was added to a final concentration of 50 mM and incubated at RT for 40 min in a dark place, followed by another 1 h incubation at 60 °C. The solution was discarded after centrifugation on a filter at 4 °C for 25 min. After adding 100 µl of 300 mM triethyl ammonium bicarbonate (TEAB), the solution was centrifuged twice for 20 min each time. After washing, TEAB and trypsin were added to the filtration unit and digested overnight for 12 h at 37°C. Finally, EAB was eluted and lyophilized. The raw data was imported into Maxquant for label-free quantitative analysis with a search engine Andromeda.

### 2.5 UPLC-QE-HF-X analysis

Mass spectrometric analysis was performed on a Q Exactive HFX mass spectrometer using a UPLC system with a BEH Amide column (2.1 mm * 100 mm, 1.7 mm). 25 mmol/L acetate of ammonium and 25 mmol/L hydroxide of ammonium in water (pH = 9.85) A) and acetonitrile B) were respectively used. The auto-sampler was set to 4 °C and the injection volume was 3 L. To obtain MS/MS spectra in information-dependent acquisition mode (IDA), a QE HFX mass spectrometer was utilized. In IDA mode, the acquisition software performed continuous evaluation of the full scan MS spectrum. Accordingly, the electrospray lonization (ESI) source conditions were as follows: 15 Arb sheath gas flow rate, 15 Arb aux gas flow rate, 350 °C capillary temperature, 60,000 full MS/MS resolution, 10/15/60 collision energy in normalized collisional energy (NCE) mode, and 3.6 (positive) or −3.2 (negative) spray voltage. The raw data was converted from XML to mzXML using ProteoWizard, and then the peak data was extracted in R using an in-house program developed by XCMS to align and integrate it. A cut-off of 0.3 was determined in metabolite annotation using an internal MS2 database.

### 2.6 Experimental animals

Male C57BL/6 mice were purchased from Jiesijie Research Institute (Shanghai, China). Six-week-old male mice (weight: 20 ± 2 g) were maintained in a standard environment at 22°C–24°C with a 12:12 h light-dark cycle, humidity (65% ± 5%) and *ad libitum* access to standardized food and tip-filtered water. Male C57BL/6 mice were given HFD (Diets, HF60, China) for 13 weeks to establish a NAFLD pathological model. Mice treated with normal chow diet (NCD) were defined as the control group. Mice fed with HFD + PPL (100, 200, and 400 mg/kg) were defined as the treatment group. Mice treated with physiological saline and PPL (400 mg/kg) were used to verify the toxicity of PPL. All experimental procedures were authorized according to the standards in the Guide for the Care and Use of Laboratory Animals. All animal experiments were approved by the Institutional Animal Care and Use Committee of Shuguang Hospital, Shanghai Chinese traditional medicine University.

### 2.7 Animals and treatment

Thirty-eight C57BL/6 male mice were randomly divided into 6 groups: 1) Normal chow diet (NCD) group (n = 6); 2) NCD + PPL (400 mg/kg) group (n = 6); 3) High fat diet (HFD) group (n = 7); 4) HFD + PPL (100 mg/kg) group (n = 6); 5) HFD + PPL (200 mg/kg) group (n = 6); 6) HFD + PPL (400 mg/kg) group (n = 7). PPL was dissolved in saline. Both NCD and HFD groups were simultaneously given saline during PPL treatment in HFD + PPL group. Mice were continuously fed HFD for 13 weeks to induce NAFLD model and treated with PPL or saline *via* oral gavage for another 6 weeks. Mice were fasted overnight, drank water freely, and anesthetized prior to specimen collection. The whole body, liver, and adipose tissues were sampled from each group of mice, weighed, and photographed for documentation. Specimens were divided into three parts, and either frozen in liquid nitrogen for proteomic studies, or stored at −80°C for biochemical analysis, or fixed in paraformaldehyde (Biosharp, BL539A, China) for further morphological and histological analysis of the liver.

### 2.8 Serum biochemical index analysis

Blood specimens were allowed to stand at RT for 30 min and then centrifuged at 4°C for 15 min at 3,000 rpm to obtain serum samples. High-density lipoprotein cholesterol (HDL-C), low-density lipoprotein cholesterol (LDL-C), serum triglycerides (TG), total cholesterols (TC), alanine aminotransferase (ALT), and aspartate aminotransferase (AST) were determined using kits (Nanjing Jian Cheng Bioengineering Institute, China) according to the manufacturer’s instructions.

### 2.9 Histopathologic analysis

Mice liver samples were fixed with 4% paraformaldehyde. White adipose tissues (WAT) and brown adipose tissue (BAT) were fixed with adipose tissue-specific fixed fluid. The liver and adipose tissue were sectioned into 5-µm sections and stained with hematoxylin and eosin (HE) solutions according to standard procedures. Slides were observed under an inverted microscope (Nikon, Ts2R-FL, Japan). Adipocyte diameter and area of white and brown adipocytes were measured using ImageJ software (National Institutes of Health, NIH, United States of America).

### 2.10 Oil red O staining

For liver sections staining, frozen liver sections were performed at optimal cutting temperature, rewarmed at RT, rinsed in distilled water, and stained with Oil Red O stain solution (Sigma, O9755, United States of America) for 6–10 min. After staining, sections were soaked in 60% isopropyl alcohol and washed with distilled water. Sections were sealed with glycerin gelatin. For cell staining, cells were harvested and fixed with 4% paraformaldehyde, washed twice, oil red O stained for 6–10 min, examined for lipid droplets. After that, samples were rinsed in 60% isopropyl alcohol and washed three times with ddH_2_O. Finally, slides were observed under an inverted microscope.

### 2.11 BODIPY fluorescence staining

For mice liver staining, liver tissue sections were frozen for 30 min and immersed in PBS for 10 min, and then soaked in BODIPY 493/503 (GLPBIO, GC42959, United States of America) for 30 min, protecting from light during staining. After washing with PBS three times, slides were counter-stained with DAPI (Beyotime Biotechnology, P0131, China). For cell staining, hepatocytes were rinsed three times with PBS and fixed in 4% paraformaldehyde for 15 min. After washing, samples were stained with BODIPY 493/503 for 30 min, and then counter-stained with DAPI for 5 min. Images were observed under an inverted microscope and fluorescence was quantified using an ImageJ software.

### 2.12 Glucose tolerance tests and insulin tolerance tests

For glucose tolerance test (GTT), mice were fasted overnight for 12 h (20:00–08:00) and drank water *ad libitum*. Fasting blood glucose levels (0 min) were measured before intraperitoneal injection of glucose (1 gkg^−1^ body weight), and blood was taken from the tail tip to record blood glucose levels (0, 15, 30, 60, and 120 min). The insulin tolerance test (ITT) was performed 3 days after the GTT test. Mice were given a 6 h fast (07:00–13:00) with free access to water, followed by an intraperitoneal injection of insulin (0.75 gkg^−1^ body weight). Blood glucose concentrations (0, 15, 30, 60, 90, and 120 min) were recorded from the tail tip.

### 2.13 Cell culture and treatment

Mouse primary hepatocytes were isolated from C57BL/6J male mice according to a two-step perfusion method as previously described (Huang et al., 2022). Briefly, cells were maintained in DMEM (Gibco, C11885500CP, United States of America) supplemented with 10% FBS (Gibco, 10100147, United States of America) plus 1% penicillin/streptomycin (Gibco, 10378016, United States of America). The immortalized normal mouse hepatocyte cell line (Alpha mouse liver 12, AML12) cell line, was inoculated in 6-well plates (2 × 10^6^ cells per well) at 37°C in a 5% CO_2_ atmosphere in DMEM/F12 medium (Meilunbio, MA0214, China) supplemented with 1% penicillin/streptomycin plus 10% FBS. The following day, both hepatocytes were washed twice and replaced with new culture medium. Blank serum metabolites, PPL metabolites (5.10 μg/ml) and 50 μM PA plus 200 μM OA were added 24 h before cell harvesting. Cells were subsequently collected for further analysis.

### 2.14 Intracellular lipid content measurement

AML12 cells and mice primary hepatocytes were inoculated in 6-well plates (2×10^6^ cells per well). After overnight incubation, cells were co-incubated with PPL metabolites (5, 10 μg/ml) and PA/OA for another 24 h. Finally, cells were collected and homogenized in lysis-buffer to determine TG content according to instructions of APPLYGEN Assay Kit.

### 2.15 Cell counting Kit-8 (CCK-8) assay

Cells were inoculated in a 96-well plate (5.0×10^3^ cells/well). Cell viability was determined using a CCK-8 assay kit (Meilunbio, MA0218-1, China). Each well was incubated with 100 μl of medium and 10 μl of CCK-8 reagent for no more than 4 h. The absorbance of each well was determined at 450 nm.

### 2.16 Quantitative real time PCR (qPCR)

Total RNA was extracted from mice liver tissues or cells using TRIzol reagent (Vazyme, R401-01, China). A total of 2 μg RNA was reversely transcribed using a HiScript^®^ II Q RT SuperMix cDNA synthesis kit (Vazyme, R233-01, China). RT-qPCR was performed using a ChamQTM Universal SYBR^®^ qPCR Master Mix (Vazyme, Q711-02, China). The relative expression levels of the genes were determined by SYBR-green-based RT-qPCR. The results were analyzed using the 2^−ΔΔCT^ method and normalized toβ-actin values. Primer sequences are listed in Supplementary Table S1.

### 2.17 Western blot

Total proteins were extracted from mice liver tissues or cells homogenized in RIPA Lysis Buffer (Beyotime, P0013B, China) supplanted with a protease inhibitor cocktail (MCE, HY-K0010, United States of America). BCA Protein Assay Reagent (Thermo Fisher Scientific, 23,225, United States of America) was used for protein concentration quantification. Approximately 40 μg of total protein samples were loaded to 10% SDS-PAGE, followed by an electrophoretic transfer to a nitrocellulose membrane. After that, the membrane was blocked with 5% BSA (Sangon biotech, A500023, China) for at RT for 1 h, and incubated at 4°C overnight against primary antibodies. After washing, membranes were incubated with secondary antibodies for 1 h. Finally, an Enhanced chemiluminescence Western blot kit (UUBIO, U10012, China) was used to visualize the target protein.

### 2.18 Histological evaluation

The pathological and morphological changes of tissue sections were assessed at ×200 magnification. The histopathological grading criteria scores of liver tissue sections include periportal-bridging necrosis, intralobular degeneration, portal inflammation, and fibrosis (Zechini B et al., 2004). The histopathological grading criteria scores of kidney tissue sections include tubular vacuolar degeneration/necrosis, tubular casts, and congestion (Gao et al., 2016). The histopathological grading criteria scores of heart tissue sections include myofibrils degeneration, oedema, subendocardial hemorrhage and the distribution of myocardial damage (Liu et al., 2021).

### 2.19 Statistical analysis

Data were expressed as the mean ± standard error of the mean (SEM). Statistical analysis was performed using GraphPad Prism software (v 8.4.2). Data were compared between groups using Student’s *t-*tests and among groups using one-way analysis of variance (ANOVA) tests. *p* < 0.05 was considered statistically significant (*p < 0.05; **p < 0.01; ***p < 0.001). If a statistically significant change was found (*p* < 0.05), a *post hoc* comparison was performed using Bonferroni correction.

## 3 Results

### 3.1 PPL retarded obesity and hepatic lipid accumulation in HFD-fed mice

**FIGURE 1:**
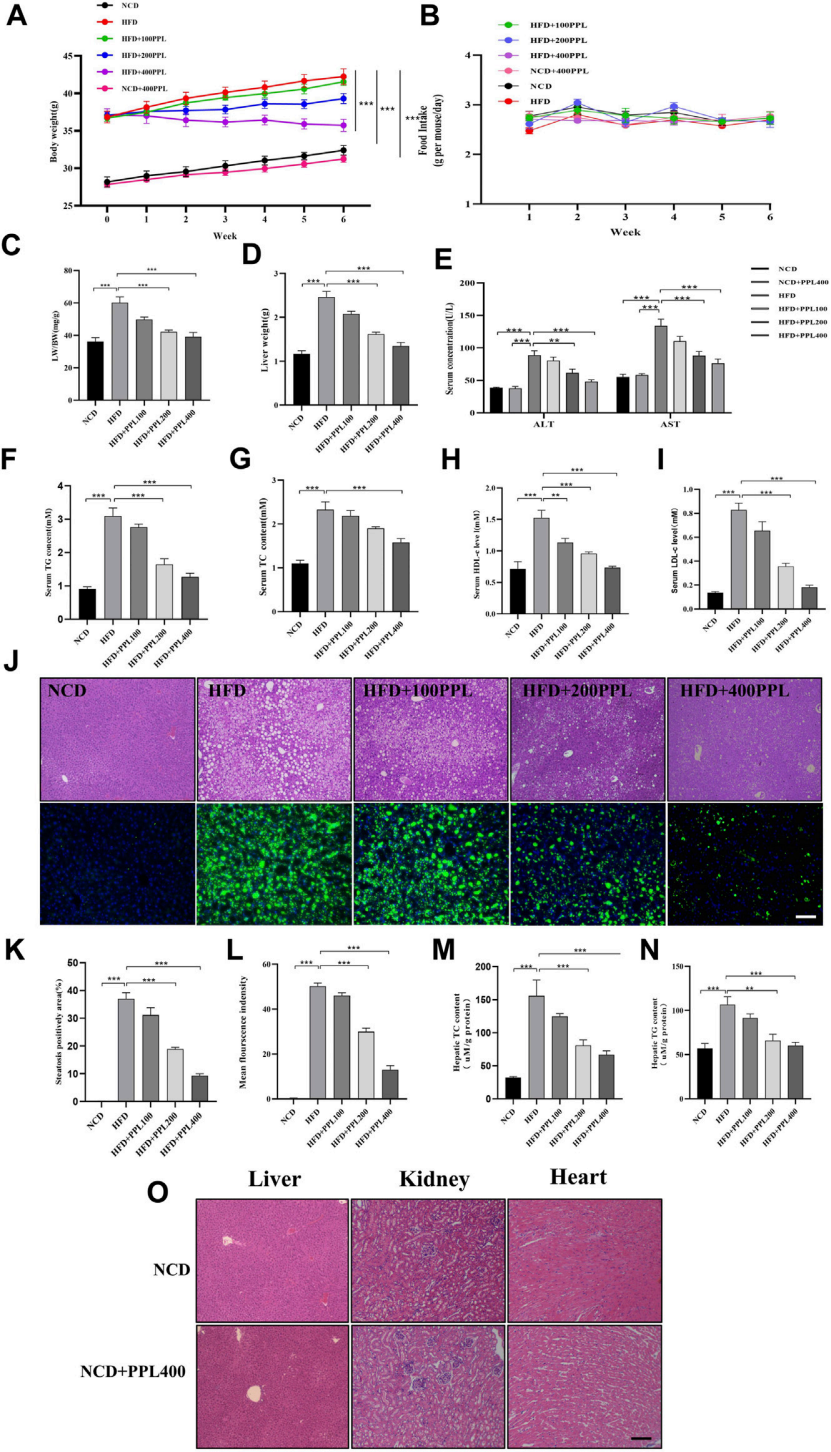
PPL alleviated HFD-induced obesity and liver steatosis in male C57BL/6 mice. **(A)** Body weight of HFD-induced obese C57BL/6 mice were fed either in the presence or absence of PPL. **(B)** Weekly food intake profile. **(C)** LW/BW ratio. **(D)** Liver weight. **(E)** Serum ALT and AST activity. **(F–I)** Serum lipid contents of TG, TC, HDL-c, and LDL-c levels. **(J)** Typical images of HE (top) and BODIPY 493/503 (bottom) staining of liver sections. **(K)** Steatosis positively area. **(L)** BODIPY positively stained area. **(M,N)** Hepatic TC content and TG content. **(O)** Typical images of HE staining from liver, kidney and heart sections. Magnification: ×200, scale bar: 100 μm. Data were expressed as the mean ± SEM. Differences among groups were compared by one-way ANOVA. *p < 0.05, **p < 0.01, ***p < 0.001. (n = 6-7 per group).

The results showed that PPL treatment significantly reversed body weight gain (Figure 1A), but no significant difference was observed in food consumption (Figure 1B). Moreover, PPL significantly reduced both liver weight (Figure 1D) and liver-to-body weight ratio (LW/BW) (Figure 1C). Long-term HFD resulted in liver dysfunction tests, as evidenced by elevated serum ALT and AST (Figure 1E), which were mitigated by PPL administration for 6 weeks. In addition, HE staining and liver histological assessment showed that PPL treatment significantly reversed the HFD-induced hepatocyte ballooning, hepatic steatosis and injury in NAFLD mice (Figure 1J). BODIPY staining further confirmed HFD-induced lipid hyper-accumulation in hepatocytes, which was reversed by PPL administration (Figure 1L). Accordingly, as indicated in Figure 1M-N, hepatic TC and TG levels were significantly elevated in HFD-fed vehicle group, while PPL treatment effectively reduced hepatic lipid content (TC, TG). Consistent with the results of hepatic lipid content measurements, PPL treatment significantly downregulated serum lipid levels in HFD-induced mice (Figures 1F–I). In addition, no significant drug toxicity was observed during PPL (400 mg/kg) treatment based on pathological assessment of the heart, liver, and kidney tissue sections (Figure 1O). Collectively, these results suggested that PPL exerted hypolipidemic activity to prevent weight gain and hinder hepatic steatosis, thereby ameliorating HFD-induced liver damage.

### 3.2 PPL relieves adipose tissue augmentation in NAFLD mice

Due to the essential role of adipocytes in the regulation of body weight and energy metabolism, increased adipocyte size caused by lipid accumulation inevitably leads to alterations in functions related to energy metabolism in different types of adipocytes. Therefore, in the present study, morphological analysis of WAT and BAT were performed in each group of mice to investigate whether the improvement in metabolic symptoms was partly attributed to a reduction in fat mass. According to WAT and BAT size measurement by HE staining, HFD-induced mice showed increased size and weight of WAT and BAT compared to NCD group, whereas PPL supplementation significantly downregulated fat mass in HFD-induced mice (Supplementary Figure S3A, S3B, S3E, S3F). Furthermore, PPL significantly suppressed gene expression levels of inflammatory factors such as *IL-6* and *MCP-1*. Accordingly, PPL significantly increased gene expression levels of browning markers such as *Tmem26*, *CD137* and *Cited1* (Supplementary Figure S3D). In addition, PPL significantly increased the expression of thermogenic markers (*PGC1α*) and significantly inhibited *PPARγ* activation, while simultaneously promoting *PPARα* expression in BAT (Supplementary Figure S3H). *GLUT4* is a major insulin-responsive glucose transporter, which is downregulated in adipose tissues in a state of insulin resistance. Here, we found that PPL significantly promoted *GLUT4* expression in both WAT and BAT (Supplementary Figure S3D, S3H). Collectively, the effect of PPL in improving glycolipid homeostasis could be partly attributed to improved adipose tissue function.

### 3.3. PPL ameliorated systemic insulin resistance and oxidative stress in obese mice

**FIGURE 2:**
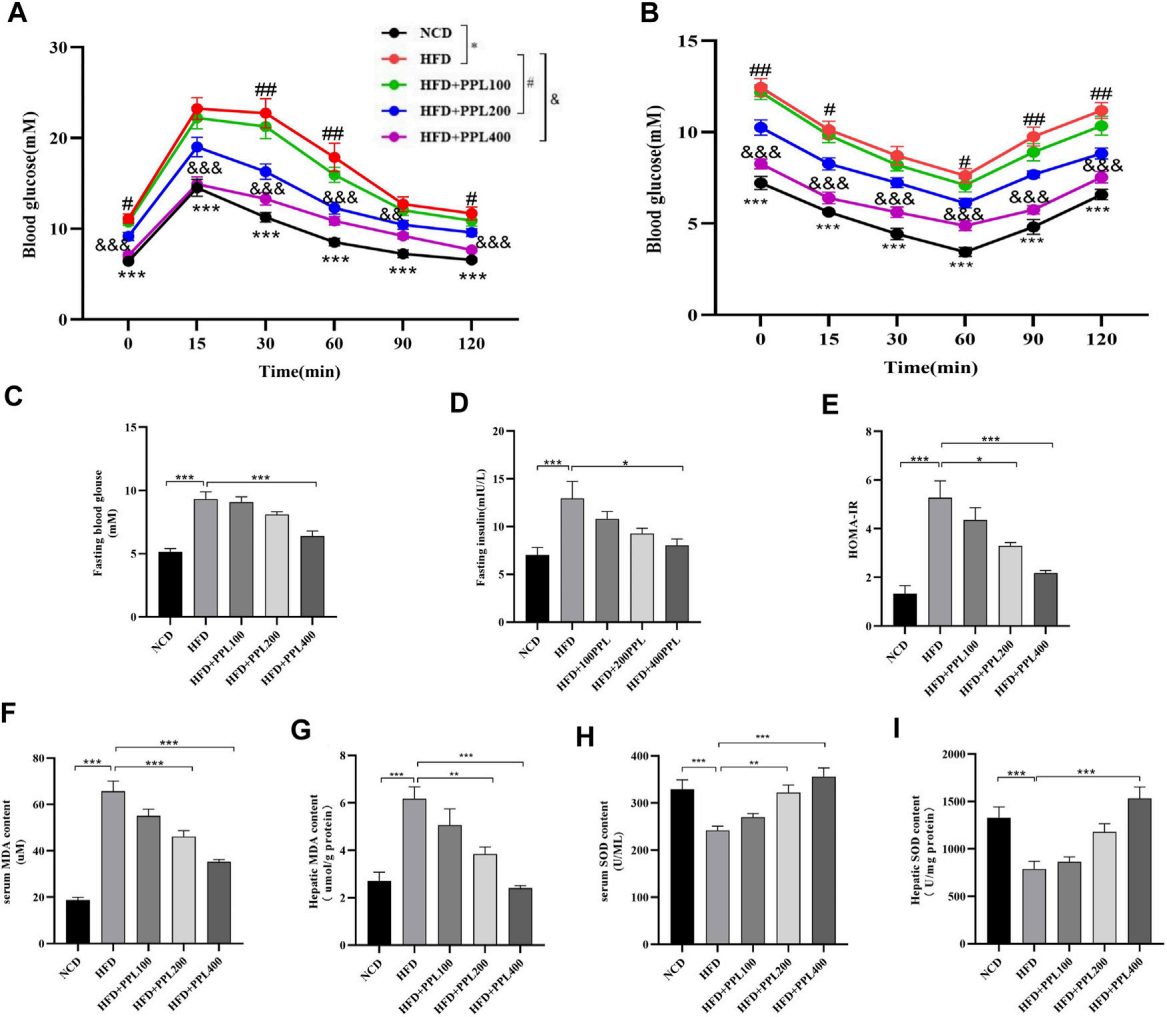
PPL improved glucose-lipid homeostasis and lipid peroxidation in HFD-induced obese mice. **(A)** Intraperitoneal injection glucose tolerance test (IPGTT). **(B)** Intraperitoneal injection insulin tolerance test (IPITT). **(C)** Fasting blood glucose. **(D)** Fasting insulin. **(E)** HOMA-IR indexes. **(F,G)** Serum and hepatic MDA content (n = 6-7 per group). **(H,I)** Serum and hepatic SOD content (n = 6-7 per group). Magnification: ×200, scale bar: 100 μm. Data were expressed as the mean ± SEM. Differences among groups were compared by one-way ANOVA. *p < 0.05, **p < 0.01, ***p < 0.001. #*p* < 0.05, ##*p* < 0.01, ###*p* < 0.001. (n = 6-7 per group).

We next investigated the effects of PPL on the regulation of systemic glucose metabolism and oxidative stress. Levels of fasting blood glucose, fasting insulin, ITTs and GTTs were measured. The results showed that PPL treatment significantly reduced HFD feeding induced higher glucose levels. Accordingly, levels of fasting blood glucose and fasting insulin (Figures 2C,D) were suppressed in PPL-fed mice compared to those detected from HFD-fed mice. In the GTT experiment, mice in the PPL200 mg/kg dose group had significantly lower blood glucose values at 0, 30, 60, and 120 min than those in the HFD-fed vehicle group. Additionally, the blood glucose levels of mice in the PPL400 mg/kg dose group at 0, 15, 30, 60, 90, and 120 min were significantly lower than those in the HFD-fed vehicle group (Figure 2A). In the ITT experiment, mice in the 200 mg/kg dose group showed significant reduction in blood glucose levels at 0, 15, 60, 90 and 120 min compared to the HFD-fed vehicle group, and mice in the 400 mg/kg dose group had significantly lower blood glucose values at 0, 15, 30, 60, 90 and 120 min than those in the HFD-fed vehicle group (Figure 2B), suggesting that PPL restored glucose homeostasis and insulin sensitivity in NAFLD mice. Next, we investigated whether PPL treatment could regulate MDA and SOD levels in NAFLD mice. The result showed that PPL significantly reduced MDA and increased SOD activity in serum and liver of NAFLD mice (Figures 2F–I). These data suggested that PPL significantly ameliorated the insulin resistance and subsequent oxidative stress in HFD-induced NAFLD mice.

### 3.4 Proteomics-based study of molecular mechanisms underlying PPL protection against NAFLD

**FIGURE 3:**
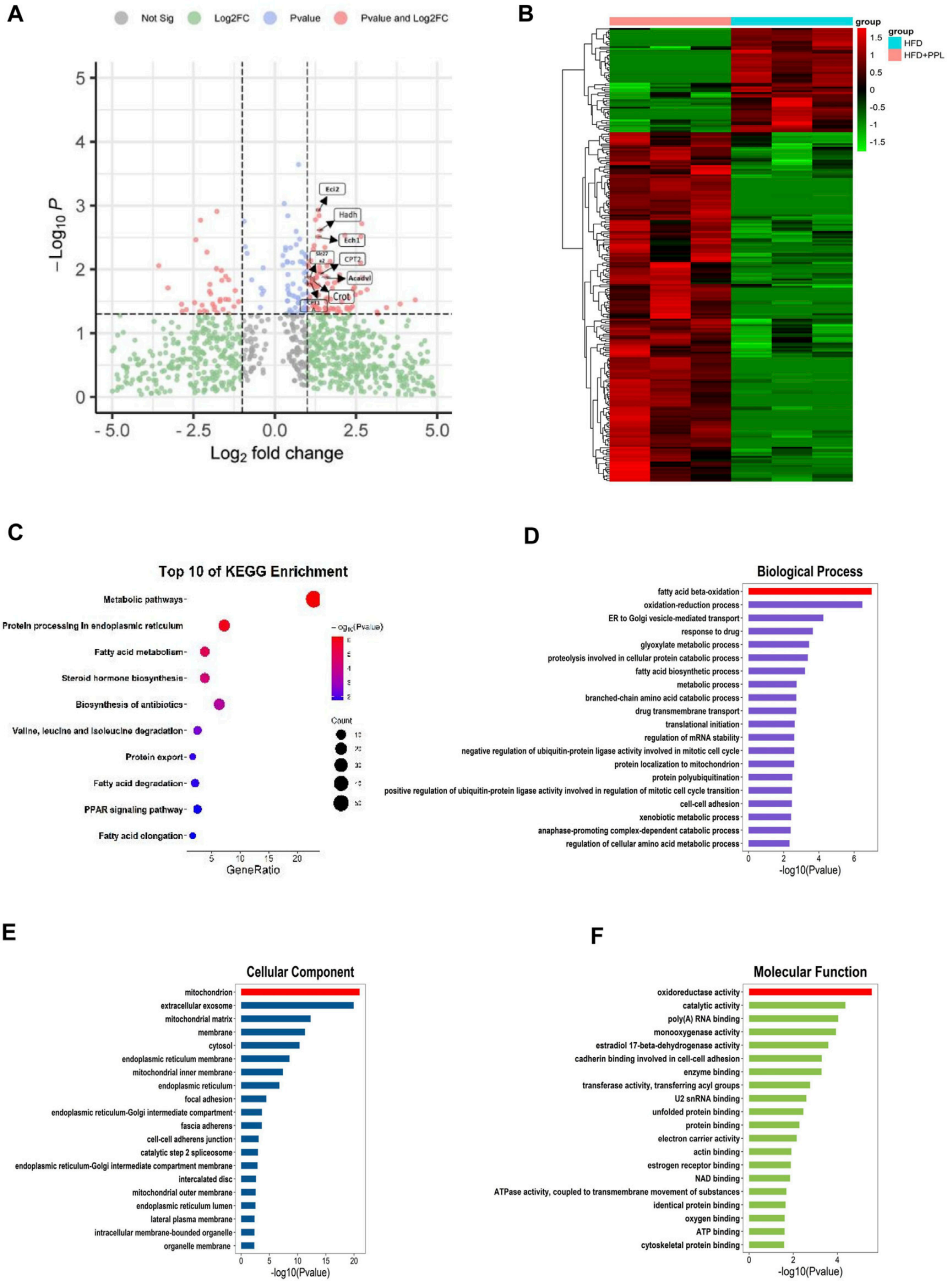
Proteomic analyses of liver specimens from differentially expressed proteins of HFD and HFD + PPL (400 mg/kg) group. **(A)** Amount of differentially expressed proteins (HFD + PPL/HFD). Upregulated, Fold change ≥1.5; downregulated, Fold change ≤0.667. FAO-related proteins were labeled. **(B)** Cluster heat map of differentially expressed proteins from HFD and HFD + PPL group. **(C)** KEGG enrichment analysis of hepatic differentially expression of proteins. **(D–F)** Biological process, Molecular function, and Cellular components for GO enrichment analysis of the differentially expressed proteins.

**FIGURE 4:**
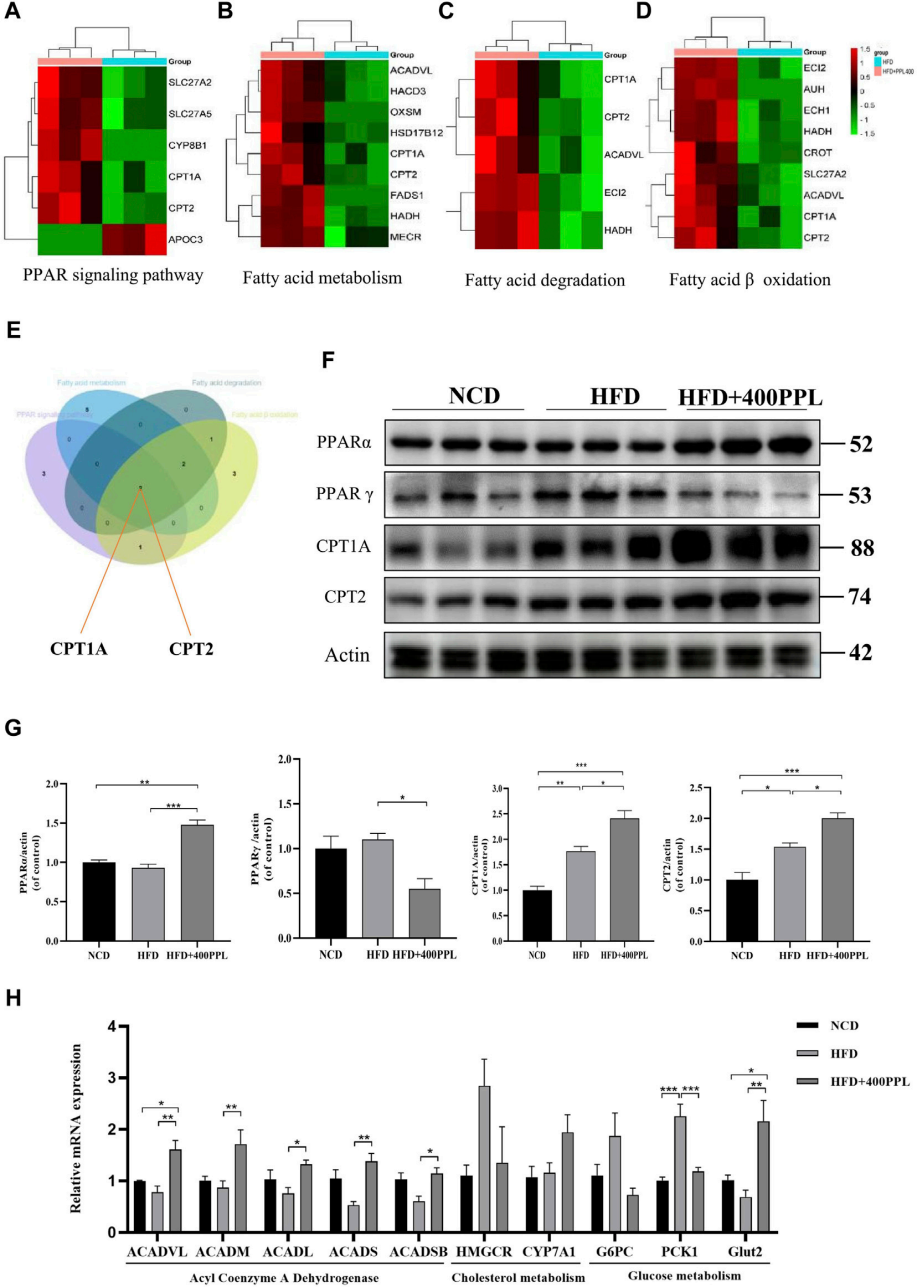
PPL improved disturbance of lipid metabolism *via* PPARs/CPT1A/CPT2-mediated FAO. **(A–D)** Differential proteins related to PPAR signal pathway, Fatty acid metabolism, Fatty acid degradation and Fatty acid β oxidation significantly upregulated and downregulated in proteomics analysis from mice treated with HFD or HFD + PPL therapy. **(E)** Key proteins between differential proteins from PPAR signal pathway, Fatty acid metabolism, Fatty acid degradation and Fatty acid β oxidation. **(F)** Expression levels of key proteins PPARα, PPARγ, CPT1A and CPT2 in PPAR signaling pathway. **(G)** Relative protein levels were compared to the NCD group after normalized to β-actin (n = 3 per group). **(H)** The relative mRNA levels related to glucose-lipid metabolism in the liver were analyzed. (n = 4-5 per group). Data were expressed as the mean ± SEM. Differences among groups were compared by one-way ANOVA. *p < 0.05, **p < 0.01, ***p < 0.001.

We further used a label-free quantitative proteomic profiling to identify the role of PPL at the hepatic tissue proteomic level to explore the key protein and molecular mechanisms underlying PPL protection against NAFLD. The protein expression profiles of liver tissues from mice in HFD and HFD + PPL (400 mg/kg) groups were subjected to by proteomic analysis, and a total of 2,693 plausible proteins were identified. A total of 249 differentially expressed proteins (DEPs) were identified by *t*-test (*p* < 0.05) in HFD and HFD + PPL groups, according to ratio analysis of protein expression levels. Of these, 191 were upregulated DEPs (FC ≥ 1.5) and 58 were downregulated DEPs (FC ≤ 0.667) (Figure 3B). KEGG pathway and GO analysis were performed using a DAVID Functional Annotation Tool. GO analysis was categorized into “biological process” (BP), “cellular compartment” (CC), and “molecular function” (MF) (Figures 3D–F). Metabolic pathways, protein processing in endoplasmic reticulum, as well as fatty acid metabolism were mostly enriched in KEGG pathways (Figure 3C), with PPAR been identified as a definitive pathway (Figure 4A). In addition, among the top 20 enrichment analysis in BP, the most affected was FAO, where oxidoreductase activity and mitochondria were identified as the most affected molecular functions and cellular components, respectively. These results suggested that PPL treatment contributed to the improvement of metabolism, fatty acid beta-oxidation, oxidoreductase activity and mitochondrial function in the HFD-fed NAFLD mice.

### 3.5 Expression profile of FAO-related proteins upregulated by PPL in liver of NAFLD mice

As previously mentioned, the most important modulator of the BP category, namely, FAO, provided us with a clear direction to further explore the potential hypolipidemic efficacy of PPL. Proteomics techniques were used to analyze the effects of PPL administration on pathway changes in liver tissues of HFD-fed mice. Total three fatty acid metabolism-related proteomes were identified, including “PPAR signaling pathway”, “Fatty acid metabolism” and “Fatty acid degradation” (Figures 4A–C). These three KEGG pathways were intercrossed FAO-associated proteins. Venn diagram analysis results showed that a pair of isozymes CPT1A and CPT2 located in the mitochondrial membrane responsible for the regulation of FAO, were simultaneously involved in the four enrichments analysis described above (Figure 4E). Based on the analysis of liver-specific proteins, we found that PPL treatment significantly inhibited the expression of the transcription factor PPARγ while promoting PPARα expression compared to the HFD group. Furthermore, in assessing the biological function of FAO in liver tissue, we found that PPL treatment significantly promoted the expression of CPT1A and CPT2, compared to the HFD group (Figure 4F). As indicated in Figures 4B–D, due to the elevated protein expression of ACADVL, we next examined the extent of transcription of multiple acyl-CoA dehydrogenases, including Acyl-CoA dehydrogenase very long chain (*ACADVL*), Acyl-CoA dehydrogenase long chain (*ACADL*), Acyl-CoA dehydrogenase medium chain (*ACADM*), Acyl-CoA dehydrogenase short chain (*ACADS*), Acyl-CoA dehydrogenase short/branched chain (*ACADSB*), which were responsible for facilitating the FAO dehydrogenation reaction. As shown in Figure 4H, PPL significantly promoted the transcriptional activities of *ACADVL*, *ACADL*, *ACADM*, *ACADS* and *ACADSB*, compared to those detected from the model group. In terms of glucose metabolism, PPL reversed the expression of gluconeogenesis-related genes (such as *G6PC* and *PCK1*) while increased the level of glucose transporter gene (*Glut2*). In addition, PPL inhibited the expression of cholesterol synthesis-related proteins (SREBP2 and HMGCR) and increased the expression level of cholesterol metabolism-related protein (CYP7A1) (Supplementary Figure S4). Taken together, these results confirmed that PPL promoted hepatic expression of signals linked with mitochondrial FAO in obese mice.

### 3.6 PPL metabolites attenuated PA/OA-induced lipid accumulation in AML12 cells

**FIGURE 5:**
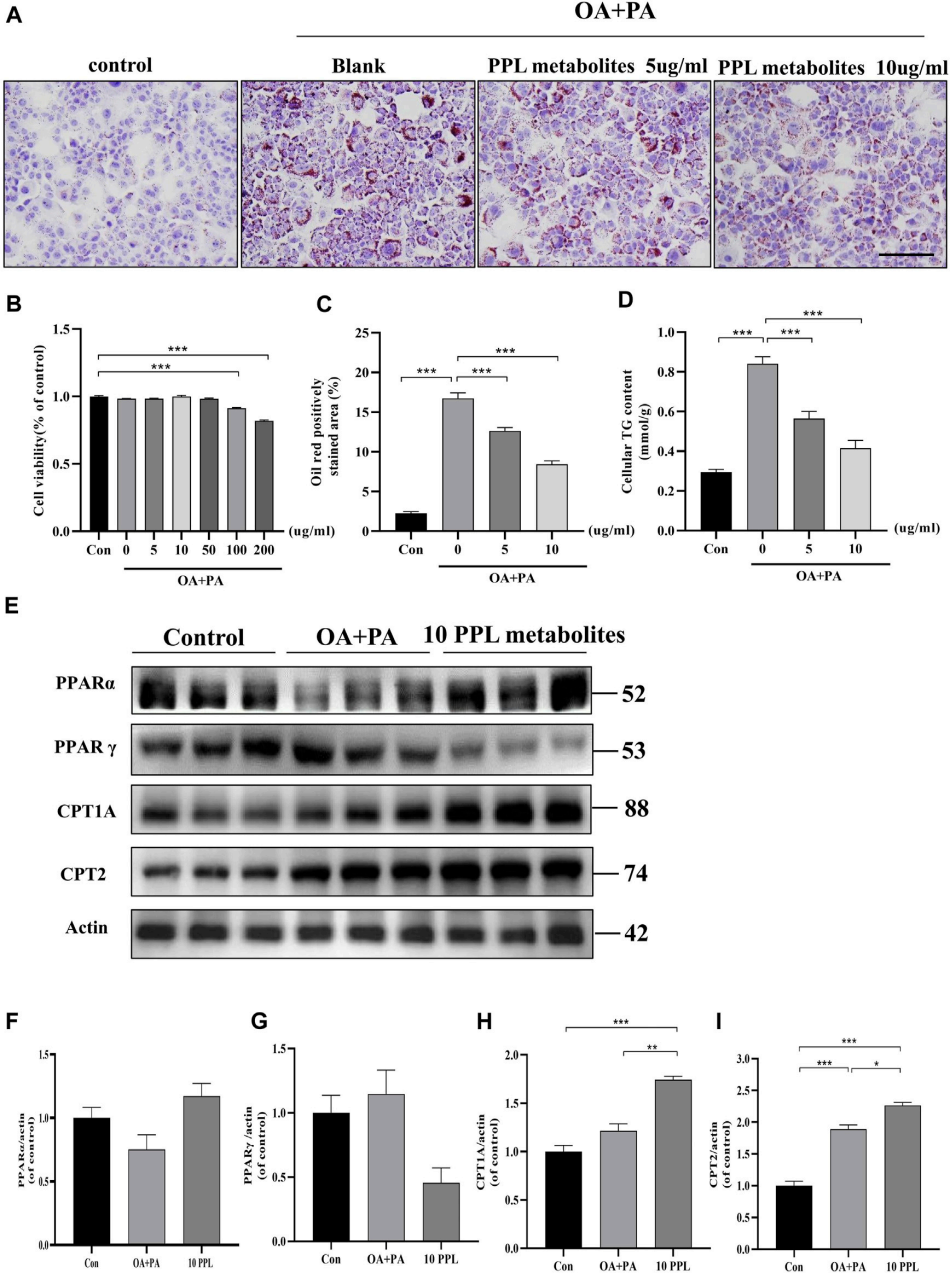
The lipid-lowering effect of PPL metabolites in PA/OA-treated AML12 cell line. **(A)** Oil Red O staining of PA/OA-induced lipid accumulation in AML12 cell line after treating with PPL metabolites. **(B)** Cell viability was detected by CCK8 after AML12 cells incubated with PPL metabolites and treated with PA/OA for 24 h (n = 9 per group). **(C)** Oil red O positively stained area was shown from each group (n = 7 per group). **(D)** Amount of cellular TG was detected from different group after above treatment (n = 4 per group). **(E)**The expression level of proteins PPARα, PPARγ, CPT1A and CPT2 in the AML12 cells were analyzed by immunoblotting (n = 3 per group). **(F–I)** The quantitative densitometric analysis of PPARα, PPARγ, CPT1A and CPT2. Magnification: ×200, scale bar: 100 μm. Data were expressed as the mean ± SEM. Differences among groups were compared by one-way ANOVA. *p < 0.05, **p < 0.01, ***p < 0.001.

*In vitro* analysis of cellular steatosis characterized by lipid accumulation was performed in AML12 cells by incubating cells with OA plus PA. The CCK-8 assay results showed no cytotoxic effects of PPL on AML12 cells at concentrations below 100 μg/ml (Figure 5B). Therefore, 5 μg/ml and 10 μg/ml of PPL metabolites were selected for subsequent *in vitro* experiments. AML12 cells were cultured in medium containing PA/OA. The Oil Red O staining results showed significant lipid deposition, whereas PPL metabolites attenuated lipid accumulation in a dose-dependent manner (Figures 5A,C). Consistent with the results described above, intracellular TG levels were significantly increased after PA/OA co-stimulation, while PPL metabolites dose-dependently downregulated intracellular TG levels (Figure 5D). Furthermore, we found that PPARγ was activated 24 h post treatment of AML12 cells with PA/OA. In contrast, PPL metabolites treatment inhibited PPARγ activation and simultaneously promoted PPARα expression. Like the results from *in vivo* assay, the downstream of transcription factor PPARα, CPT1A and CPT2 were significantly upregulated (Figure 5E). These results suggested that alterations in lipid content induced by PPL metabolites treatment in AML12 cell were mediated *via* an effect on FAO.

### 3.7 PPL metabolites reduced PA/OA-exposed fat accumulation in mice primary hepatocytes

**FIGURE 6:**
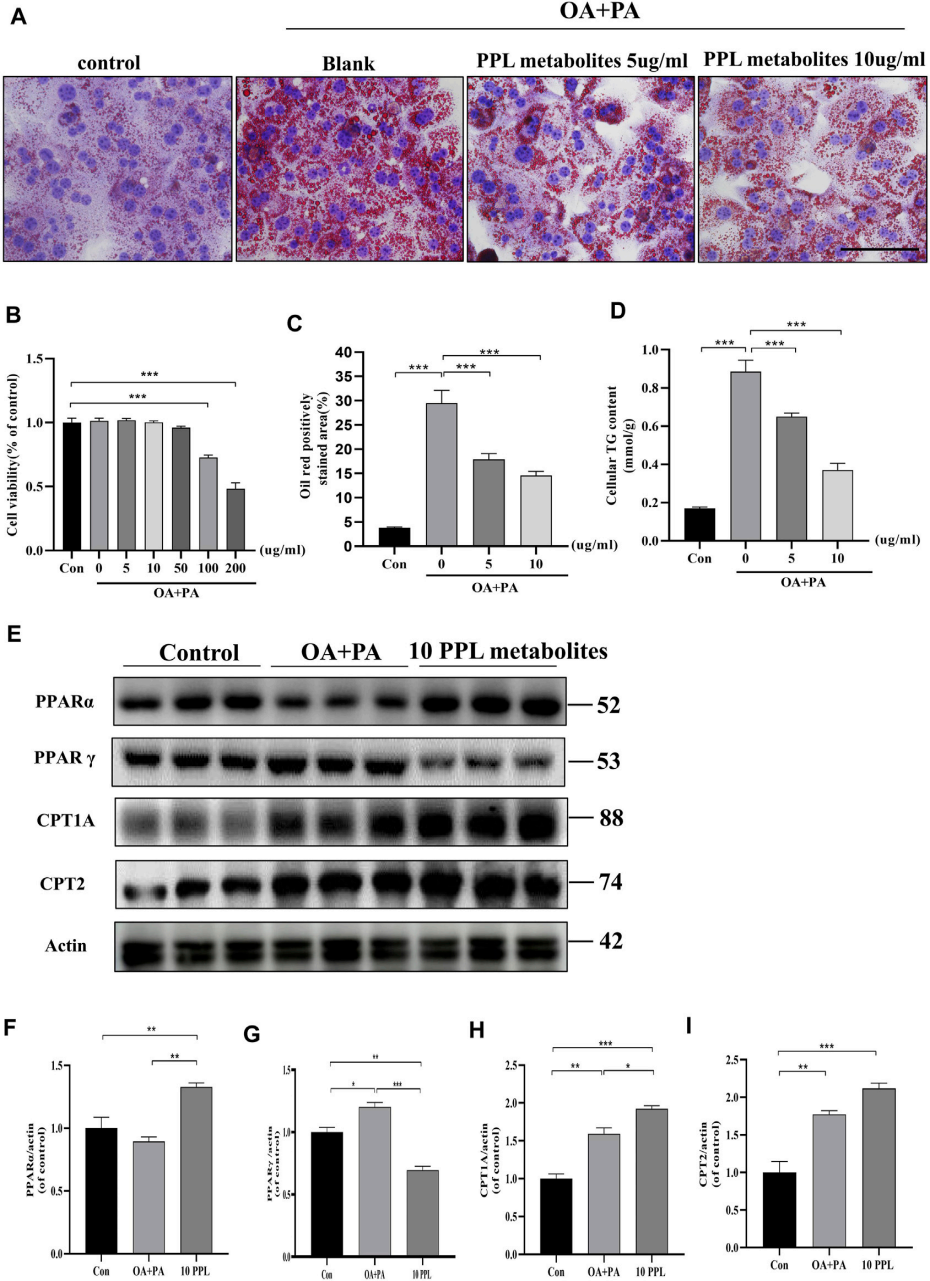
The anti-lipid deposition effect of PPL metabolites in PA/OA-treated mice primary hepatocytes. **(A)** Oil Red O staining of PA/OA-induced lipid accumulation in primary hepatocytes after treating with PPL metabolites. **(B)** Cell viability was detected by CCK8 after primary hepatocytes incubated with PPL metabolites and treated with PA/OA for 24 h (n = 10 per group). **(C)** Oil red O positively stained area was shown from each group (n = 7 per group). **(D)** Amount of cellular TG was detected from different group after treatment (n = 4 per group). **(E)** The expression level of proteins PPARα, PPARγ, CPT1A and CPT2 in the primary hepatocytes were analyzed by immunoblotting (n = 3 per group). **(F–I)** The quantitative densitometric analysis of PPARα, PPARγ, CPT1A and CPT2. Magnification: ×400, scale bar: 100 μm. Data were expressed as the mean ± SEM. Differences among groups were compared by one-way ANOVA. *p < 0.05, **p < 0.01, ***p < 0.001.

Similarly, we examined the hypolipidemic effect of PPL metabolites on mice primary hepatocytes. CCK-8 analysis results (Figure 6B) indicated that PPL metabolites (5, 10, 50 μg/mL) showed no significant effect on the cell viability of mice primary hepatocytes. Accordingly, 5 μg/ml and 10 μg/ml were selected as the optimum dosing concentrations for subsequent experiments. As indicated in Figures 6A–C, PPL metabolites (5, 10 μg/ml) significantly reversed PA/OA-induced cellular lipid accumulation, which was consistent with the results in Figure 6D that PPL (5, 10 μg/ml) reduced the PA/OA-induced elevated cellular TG levels in mice primary hepatocytes. To further investigate the effect of PPL metabolites on FAO and to identify potential molecular functions involved, we tested whether PPL metabolites significantly reversed the activation of PPARγ, a transcription factor responsible for fat storage. Meanwhile, PPL metabolites significantly promoted the activity of PPARα, a nuclear receptor responsible for lipolysis. Compared to the model group, downstream proteins CPT1A and CPT2 were also upregulated (Figure 6E). These results demonstrated that PPL metabolites alleviated lipid accumulation through affecting FAO in mice primary hepatocytes.

### 3.8 Fatty acid oxidation inhibitor blocked the lipid-lowering phenotype of PPL metabolites in PA/OA-treated AML12 cells and mice primary hepatocytes

**FIGURE 7:**
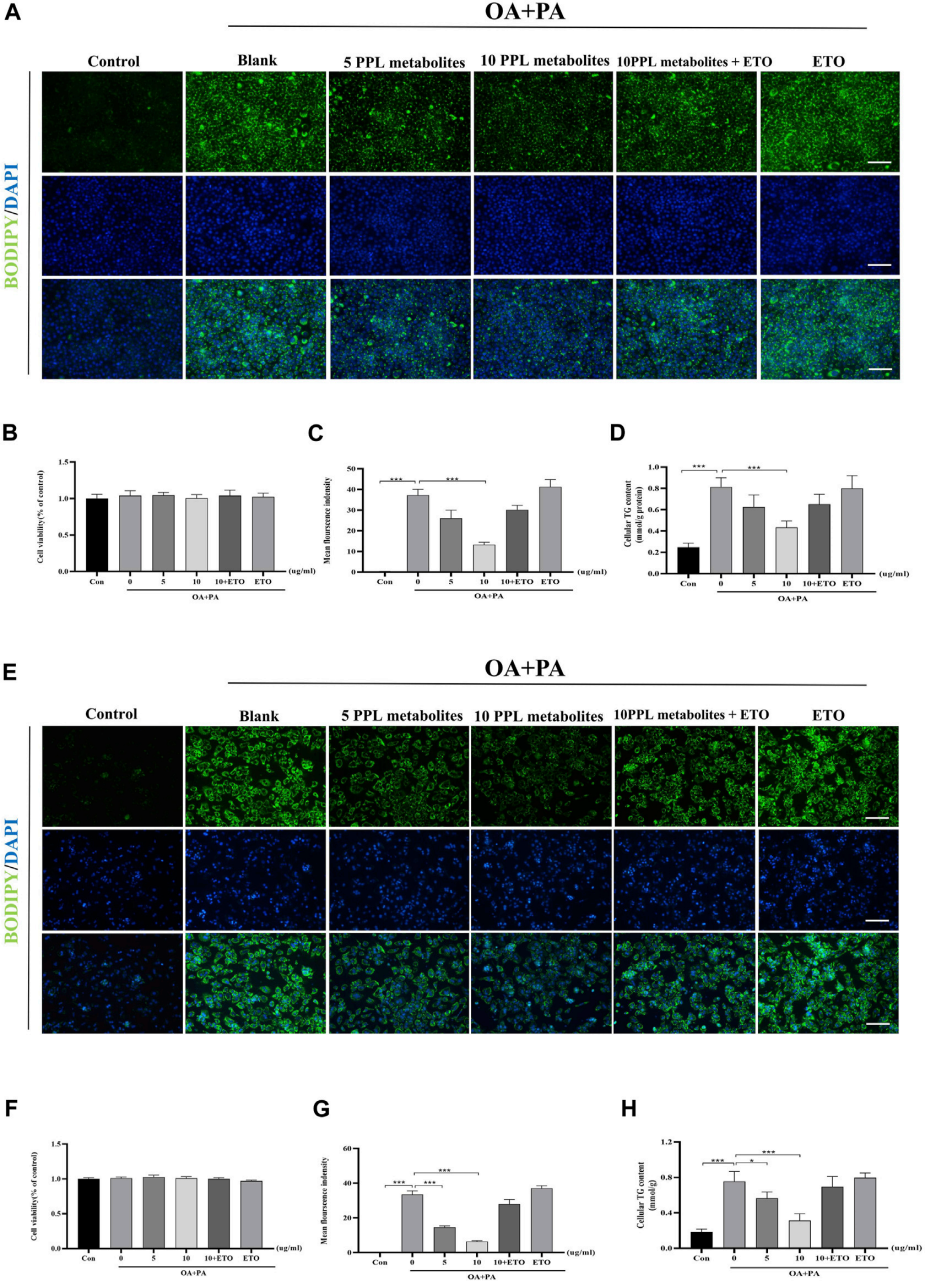
ETO blocked the lipid-lowering effect of PPL metabolites in PA/OA-treated hepatocytes. **(A)** BODIPY 493/503 staining of PA/OA-induced lipid accumulation in AML12 cells after treating with PPL metabolites and ETO for 24 h. **(B)** Cell viability was detected by CCK8 assay after AML12 cells incubated with PPL metabolites and ETO for 24 h (n = 9 per group). **(C)** BODIPY positively stained area (n = 3 per group). **(D)** Cellular TG levels were detected after AML12 cells incubated with PPL metabolites and ETO for 24 h (n = 4 per group). **(E)** BODIPY 493/503 staining of PA/OA-induced lipid accumulation in mice primary hepatocytes after treated with PPL metabolites and ETO for 24 h. **(F)** Cell viability was detected by CCK8 after mice primary hepatocytes incubated with PPL metabolites and ETO for 24 h (n = 9 per group). **(G)** BODIPY positively stained area (n = 4 per group). **(H)** Cellular TG levels were detected after mice primary hepatocytes incubated with PPL metabolites and ETO for 24 h (n = 4 per group). Magnification: ×200, scale bar: 100 μm. Data were expressed as the mean ± SEM. Differences among groups were compared by one-way ANOVA. *p < 0.05, **p < 0.01, ***p < 0.001.

ETO is a fatty acid oxidation inhibitor that inhibits the activity of CPT1A enzyme through binding to its catalytic site and thus has been widely used to validate the molecular function of the hypolipidemic activity of PPL metabolites *in vitro*. The CCK-8 assay showed that PPL metabolites (5, 10 μg/ml) + ETO (50 μM) did not affect the cell viability of hepatocytes (Figures 7B, F). Accordingly, PPL (10 μg/ml) + ETO (50 μM) was used for the following experiments. To assess lipid content more accurately, an alternative lipid dye BODIPY 493/503, which has a higher sensitivity for lipid droplets, was applied. The BODIPY staining demonstrated that PPL metabolites attenuated lipid droplets in a dose-dependent manner, whereas ETO (50 μM) reversed lipid-lowering phenotype of PPL metabolites (10 μg/ml) compared to PA/OA-treated AML12 cells (Figure 7A). Consistent with the results of fluorescence staining of AML12 cells, the anti-lipid capacity of PPL (10 μg/ml) was blocked by co-incubation with ETO in OA/PA-treated mice primary hepatocytes (Figures 7E,G,H). These results suggested that PPL metabolites blocked lipid accumulation through affecting FAO in AML12 cells and mice primary hepatocytes.

### 3.9 Identification and analysis of components in PPL metabolites

To explore the material basis of PPL for ameliorating HFD-induced NAFLD, UPLC/QE-HFX analysis was used. The results showed that total 29 compounds were identified from PPL metabolites (Supplementary Figure S1 and S2). Of these, 12 compounds (Quercetin, Kaempferol, Genistein, Baicalin, Phloretin, Hydroxygenkwanin, Baicalein, Galangin, Apigenin, Apigenin 7-O-glucuronide, M1 and M2) were flavonoids; 1 compound (Emodin) was quinones, four compounds (4-Hydroxybenzoic acid, 4-hydroxybenzaldehyde, Para-cresol, and Protocatechualdehyde) were phenols, threecompounds (Chlorogenic acid, Praeruptorin A and Demethylwedelolactone) were phenylpropanoids, four compounds (3-O-Acetyldiosgenin, Cholic acid, Ecliptasaponin A and Deoxycholic Acid) were terpenoids, one compound (Abscisic acid) was sesquiterpenoids, two compounds (Guanine and Nicotinic acid) were alkaloids, two compounds (Isatin and Fumaric acid) were derivatives, and two compounds (Melibiose and Isobutyl 4-hydroxybenzoate) were others (Supplementary Table S1 and Supplementary Table S2). Taken together, it was suggested that the main component of PPL metabolites were flavonoids, which might play a major pharmacological activity in anti-NAFLD efficacy of PPL.

## 4 Discussion

Obesity is one of the main causes of NAFLD worldwide, accompanied by hyperlipidemia, hypertension, and hyperglycemia, and its pathological roots lie in the imbalance between caloric intake and energy expenditure (Polyzos et al., 2019). Excess lipid accumulation interferes with the physiological properties of normal hepatocytes, which store large amounts of glycogen and small amounts of lipids, resulting in dysfunctional hepatic lipid and carbohydrate metabolism (Stanhope, 2016; Petersen et al., 2017). The therapeutic strategies for NAFLD mainly focus on reducing lipid synthesis, enhancing fatty acid catabolism, and improving mitochondrial FAO to restore metabolic homeostasis (Postic et al., 2008; Ipsen et al., 2018).

In recent years, many studies have shown that numerous plant metabolites or monomers therein can exert different combinations of biological activities to ameliorate weight gain as well as reverse hepatic steatosis (Yan et al., 2020; Dai et al., 2021; Zhou et al., 2021). Previous studies have demonstrated that PPL exerted hepatoprotection in acute alcoholic liver injury by scavenging lipid peroxides and enhancing antioxidant properties (Wei et al., 2016). Furthermore, similar hepatoprotective effects were replicated in dimethyl nitrosamine (DMN)-induced liver fibrosis, and the biological function of this phenotype identified that PPL restraining the transformation of hepatic stellate cells to fibroblasts (Cao et al., 2017). However, the specific role and regulatory mechanisms of PPL in ameliorating HFD-induced hepatic steatosis and injury remain elusive. In this study, we found that the traditional Chinese medicine PPL significantly ameliorated overweight, fasting insulin, hyperlipemia and improved hepatic steatosis in mice, without significant effect on their food intake. These results suggested that PPL enhanced insulin sensitivity and restored glucose and lipid metabolic homeostasis in HFD-induced obese mice.

**FIGURE 8:**
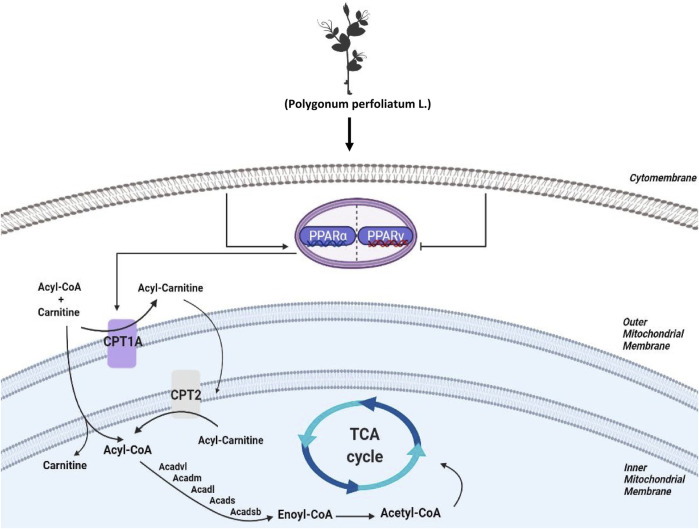
Schematic diagram depicting the role of FAO in the lipid-lowing effects of PPL. PPL significantly suppressed the expression of transcription factors PPARγ, promoted the transcriptional level of PPARα, and significantly activated CPT1A and CPT2 as well as a series of Acetyl-Coenzyme A dehydrogenase that promote fatty acid β-oxidation, thus ameliorating obesity, NAFLD and insulin resistance.

To investigate the biology functions of PPL blocking high fat-treated obesity and NAFLD, proteomic analysis was conducted in this study. Compared to the HFD group, we found that FAO was the most affected biological process in mice liver tissues from PPL group. Among the FAO-related proteins, CPT1A and CPT2 were found to be simultaneously involved in PPAR signaling pathway, fatty acid metabolism, and fatty acid degradation, suggesting that the pair of isozymes located in the inner and outer mitochondrial membranes functions a key role in the inhibition of NAFLD. In addition, ACADVL was also detected, which encodes a dehydrogenase that catalyzes the first step in physiological β-oxidation of fatty acid to generate Enoyl-CoA (Figure 8). Accordingly, we examined a series of hepatic dehydrogenase genes and found that the transcriptional activities of different dehydrogenases were substantially much lower in the model group than in the PPL treated group.

Mitochondrial FAO is the primary pathway of fatty acid catabolism and plays a crucial role in the regulation of daily energy homeostasis (Knottnerus et al., 2018). According to pathophysiology, FAO is closely associated with numerous diseases such as obesity, cancer, heart failure, and skeletal myopathy (Fillmore et al., 2014; Qu et al., 2016; Fucho et al., 2017; Pennisi et al., 2018). As key isoenzymes of FAO, CPT1 and CPT2 both regulate energy metabolism in normal physiological and disordered status (Bonnefont et al., 2004). The CPT1 protein family includes three tissue-specific isoforms: “liver” (CPT1A), “brain” (CPT1-C), and “muscle” (CPT1B) (Bonnefont et al., 2004; Schlaepfer and Joshi, 2020). CPT2 is a substrate that acts as a mitochondrial long-chain FAO to convert acylcarnitine to acyl-CoA in mitochondria (Houten et al., 2020). In this study, the protein expression of both CPT1A and CPT2 was significantly upregulated in liver tissues of PPL-treated mice relative to the obese mice. In short, our results suggested that PPL notably activated hepatic FAO *in vivo*.

In this study, flavonoid components were found to be probably the main components of anti-NAFLD in PPL. Among them, kaempferol, genistein, quercetin and phloretin can improve NAFLD through PPAR signaling pathway (Chang et al., 2011; Alsanea et al., 2017; Davis and Hastings, 2018; Lv et al., 2019; Wang et al., 2021). Both baicalin and phloretin enhance FAO as an approach to promote fatty acid inflow by increasing the activity of CPT1A (Alsanea et al., 2017; Dai et al., 2018). Apigenin and baicalein can sensitize the autophagy/AMPK pathway to restore lipid metabolism (Zhang et al., 2019; Sun et al., 2020). Therefore, the above results have provided a theoretical basis for the hypolipidemic effect of PPL against NAFLD.

Previous results have shown that various flavonoids in PPL are able to reduce lipid accumulation *in vitro*, we therefore hypothesized that PPL could play a similar role in PA/OA-stimulated hepatocyte models. Subsequently, immortalized hepatocytes, AML12 cells, which retain representative hepatocyte characteristics, were used as a suitable cell line in this study. The results showed that PPL significantly reversed the elevated TG levels in PA/OA-stimulated AML12 cells, presumably associated with the upregulation of CPT1A, CPT2 and PPARα protein expression levels. In addition, elevated FAO, further inhibited PPARγ protein expression to block lipid storage. However, the FAO inhibitor ETO significantly reversed the hypolipidemic effect delivered from PPL metabolites in PA/OA-treated hepatocytes and replicated a similar phenotype in primary mice hepatocytes. All these results suggested that the hypolipidemic effect of PPL might be related to the promotion of mitochondrial β-oxidation, implying FAO might be an attractive pharmacological mechanism for explaining PPL treatment against NAFLD.

In summary, our results demonstrated that PPL, a traditional Chinese medicine, could effectively ameliorate HFD-induced hyperlipidemia, abnormal liver function, lipid peroxidation, as well as hepatic steatosis. This efficacy was achieved by promoting mitochondrial FAO through PPARs/CPT1A/CPT2 pathway. Furthermore, flavonoids might be the major active ingredients in PPL. Our findings have indicated a prospect for the development of PPL and its flavonoids in clinical treatment of NAFLD.

## Data Availability

The datasets presented in this study can be found in online repositories. The names of the repository/repositories and accession number(s) can be found below: http://www.proteomexchange.org/, PXD033219.

## Nomenclature

### Abbreviations

(NAFLD): Non-alcoholic fatty liver disease
(PPL): Polygonum perfoliatum L
(HFD): high fat diet
(FAO): fatty acid β oxidation
(CPT1A): Recombinant Carnitine Palmitoyltransferase 1A
(CPT2): Recombinant Carnitine Palmitoyltransferase 2
(PPARs): peroxisome proliferators-activated receptors
(ETO): Etomoxir
(UPLC/QE-HFX): Ultra-high-performance liquid chromatography/Q Exactive-HFX
(acyl-CoA): acyl coenzyme A
(acetyl-CoA): acetyl coenzyme A
(MDA): Malondialdehyde
(SOD): Superoxidedismutase
(TC): total cholesterol
(TG): Triglyceride
(PMSF): Phenylmethanesulfonyl fluoride
(FASP): filter-aided sample preparation
(TEAB): triethyl ammonium bicarbonate
(IDA): information-dependent acquisition mode
(NCE): normalized collisional energy
(FASP): filter-aided sample preparation
(ESI): electrospray lonization
(HDL-C): High density lipoprotein cholesterol
(LDL-C): low density lipoprotein cholesterol
(ALT): alanine aminotransferase
(AST): aspartate aminotransferase
(WAT): white adipose tissues
(BAT): brown adipose tissue
(HE): hematoxylin and eosin
(AML12): Alpha mouse liver 12
(GTT): glucose tolerance test
(ITT): insulin tolerance test
(SEM): standard error of the mean
(ACADVL): Acyl-CoA dehydrogenase very long chain
(ACADL): Acyl-CoA dehydrogenase long chain
(ACADM): Acyl-CoA dehydrogenase medium chain
(ACADS): Acyl-CoA dehydrogenase short chain
(ACADSB): Acyl-CoA dehydrogenase short/branched chain
(DMN): dimethyl nitrosamine
