# Supplementary material for: Quantitative proteomics reveals Polygonum perfoliatum L. ameliorates hepatic steatosis by promoting PPARs/CPT1A/CPT2-mediated fatty acid β-oxidation
Source: Front Pharmacol. 2023 Mar 23;14:1016129. doi: 10.3389/fphar.2023.1016129 (PMC10076547; doi:10.3389/fphar.2023.1016129)
Supplement: Supplementary file 1 [file DataSheet1.docx]

**Table S1. Primer for quantitative real-time PCR detection**

| Gene | Primer Forward | Primer Reverse |
| --- | --- | --- |
| Acadm | TCAGAGTGCCTAAGGAAAATGT | CGACTGTAGGTCTGGTTCTATC |
| Acads | GATTACCTGGCCTACTCCATC | TAGAGAGAATTGTTGACGCTCA |
| Acadl | AAACAGTTGCACACATACAGAC | ATTCAGATGCCCAGTATTTTGC |
| Acadvl | GATGCTTCCACCAGAGAAAAAC | GAACACCTGATCAATGGTAAGC |
| Acadsb | ATTACTACGTCCTCAATGGGTC | ACCTTCTGTATCTCGGTCTACT |
| IL-1β | GCAACTGTTCCTGAACTCAACT | ATCTTTTGGGGTCCGTCAACT |
| IL-6 | TAGTCCTTCCTACCCCAATTTCC | TTGGTCCTTAGCCACTCCTTC |
| MCP-1 | TTAAAAACCTGGATCGGAACCAA | GCATTAGCTTCAGATTTACGGGT |
| UCP-1  Tmem26  CD137  Cited1  Glut4  Prdm16  PPARα  PPARγ  PGC1α  Cidea | TGGTGAACCCGACAACTTCC  TTCCTGTTGCATTCCCTGGTC  CGTGCAGAACTCCTGTGATAAC  AACCTTGGAGTGAAGGATCGC  GTGACTGGAACACTGGTCCTA  CCAAGGCAAGGGCGAAGAA  AGAGCCCCATCTGTCCTCTC  TCGCTGATGCACTGCCTATG  TATGGAGTGACATAGAGTGTGCT  TGACATTCATGGGATTGCAGAC | GGCCTTCACCTTGGATCTGAA  GCCGGAGAAAGCCATTTGT  GTCCACCTATGCTGGAGAAGG  GTAGGAGAGCCTATTGGAGATGT  CCAGCCACGTTGCATTGTAG  AGTCTGGTGGGATTGGAATGT  ACTGGTAGTCTGCAAAACCAAA  GAGAGGTCCACAGAGCTGATT  CCACTTCAATCCACCCAGAAAG  GGCCAGTTGTGATGACTAAGAC |
| PCK1  G6PC | CTGCATAACGGTCTGGACTTC  CGACTCGCTATCTCCAAGTGA | CAGCAACTGCCCGTACTCC  GTTGAACCAGTCTCCGACCA |
| HMGCR  SREBP2  CYP7A1  Glut2 | AGCTTGCCCGAATTGTATGTG  CAGGTGCAGACGGTACAGG  GGGATTGCTGTGGTAGTGAGC  ATCGCTCCAACCACACTCAG | TCTGTTGTGAACCATGTGACTTC  CGACCCTTACTGGCACTTGAA  GGTATGGAATCAACCCGTTGTC  GCTGAGGCCAGCAATCTGAC |

**Table S1. UPLC/QE-HFX data of the identified components in PPL metabolizes**

| **Chemical structure** | **NameEN** | **Formula** | **mzmed** | **rtmed** | **Class** |
| --- | --- | --- | --- | --- | --- |
| 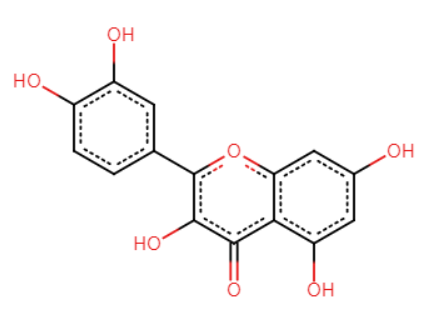   \|  \| \| --- \| \| | Quercetin | C15H10O7 | 303.05013 | 542.467 | Flavonoids |
|  |  |  |  |  |  |
|  |  |  |  |  |  |
|  |  |  |  |  |  |
| 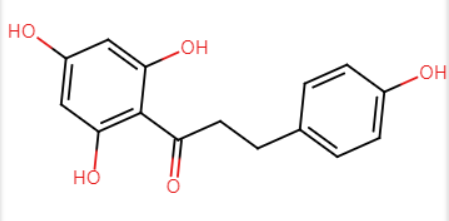   \|  \| \| --- \| \| | Phloretin | C15H14O5 | 275.09167 | 478.402 | Flavonoids |
|  |  |  |  |  |  |
|  |  |  |  |  |  |
|  |  |  |  |  |  |
| 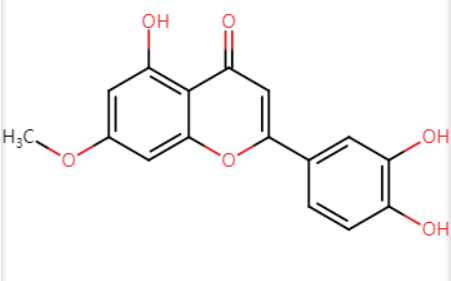   \|  \| \| --- \| \| | Hydroxygenkwanin | C16H12O6 | 301.06088 | 413.429 | Flavonoids |
|  |  |  |  |  |  |
|  |  |  |  |  |  |
|  |  |  |  |  |  |
| 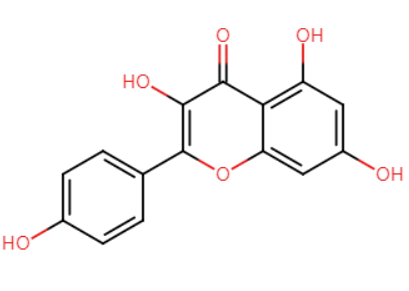   \|  \| \| --- \| \| | Kaempferol | C15H10O6 | 287.05513 | 482.3505 | Flavonoids |
|  |  |  |  |  |  |
|  |  |  |  |  |  |
|  |  |  |  |  |  |
| 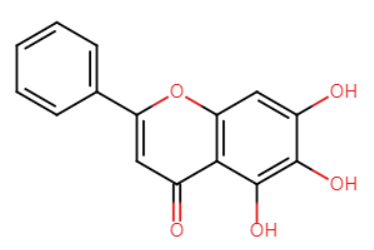   \|  \| \| --- \| \| | Baicalein | C15H10O5 | 271.05974 | 613.012 | Flavonoids |
|  |  |  |  |  |  |
|  |  |  |  |  |  |
|  |  |  |  |  |  |
| 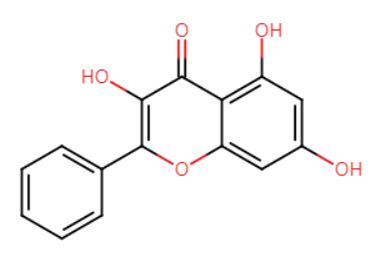   \|  \| \| --- \| \| | Galangin | C15H10O5 | 271.06008 | 582.58 | Flavonoids |
|  |  |  |  |  |  |
|  |  |  |  |  |  |
|  |  |  |  |  |  |
| 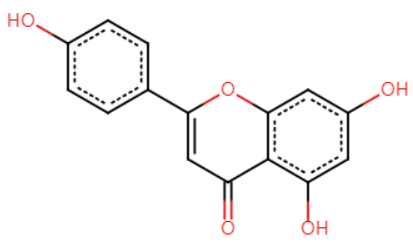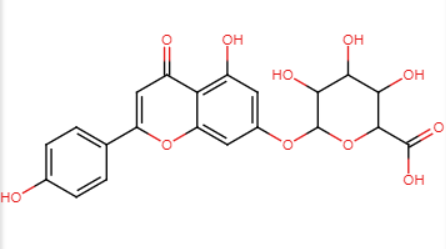   \|  \| \| --- \| \| | Apigenin | C15H10O5 | 271.06016 | 548.756 | Flavonoids |
|  |  |  |  |  |  |
|  |  |  |  |  |  |
|  |  |  |  |  |  |
| 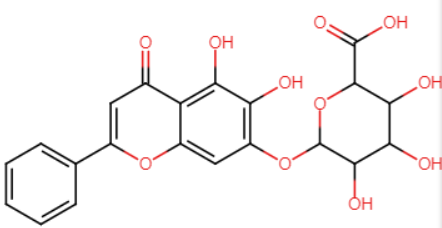   \|  \| \| --- \| \| | Apigenin 7-O-glucuronide | C21H18O11 | 447.09291 | 724.947 | Flavonoids |
|  |  |  |  |  |  |
|  |  |  |  |  |  |
|  |  |  |  |  |  |
|  | Baicalin | C21H18O11 | 445.07803 | 780.213 | Flavonoids |
|  |  |  |  |  |  |
|  |  |  |  |  |  |
|  |  |  |  |  |  |
| 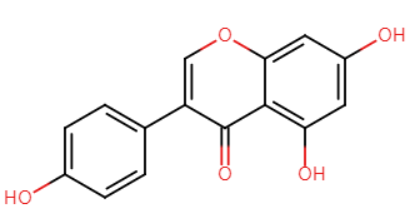   \|  \| \| --- \| \| | Genistein | C15H10O5 | 269.04531 | 616.3785 | Flavonoids |
|  |  |  |  |  |  |
|  |  |  |  |  |  |
|  |  |  |  |  |  |
| 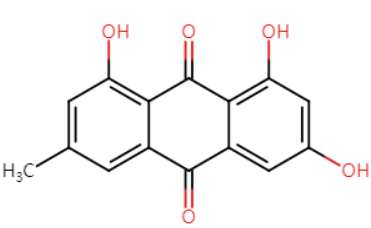   \|  \| \| --- \| \| | Emodin | C15H10O5 | 271.06007 | 600.1755 | Quinones |
|  |  |  |  |  |  |
|  |  |  |  |  |  |
|  |  |  |  |  |  |
| 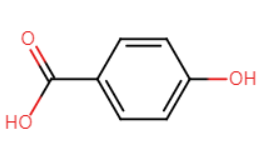   \|  \| \| --- \| \| | 4-Hydroxybenzoic acid | C7H6O3 | 137.02448 | 156.247 | Phenols |
|  |  |  |  |  |  |
|  |  |  |  |  |  |
|  |  |  |  |  |  |
| 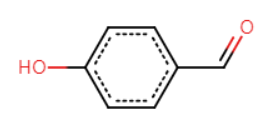   \|  \| \| --- \| \| | 4-hydroxybenzaldehyde | C7H6O2 | 121.02946 | 196.204 | Phenols |
|  |  |  |  |  |  |
|  |  |  |  |  |  |
|  |  |  |  |  |  |
| 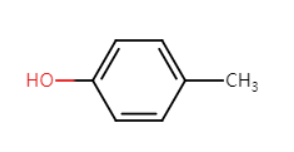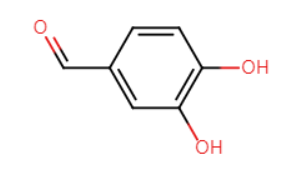   \|  \| \| --- \| \| | Para-cresol | C7H8O | 107.04979 | 265.985 | Phenols |
|  |  |  |  |  |  |
|  |  |  |  |  |  |
|  |  |  |  |  |  |
| 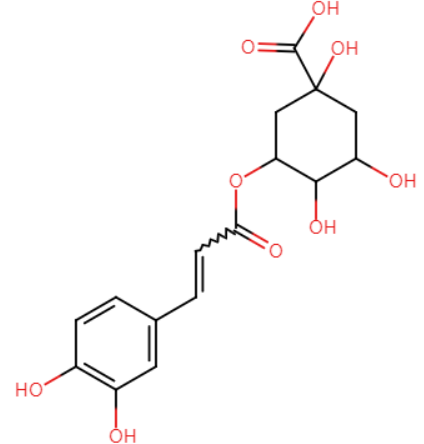   \|  \| \| --- \| \| | Protocatechualdehyde | C7H6O3 | 137.02441 | 754.317 | Phenols |
|  |  |  |  |  |  |
|  |  |  |  |  |  |
|  |  |  |  |  |  |
|  | Chlorogenic acid | C16H18O9 | 353.0882 | 615.0795 | Phenylpropanoids |

**Figure S1**

**UPLC/QE-HFX base peak intensity chromatograms of PPL extract (B) and PPL metabolites (C) in negative mode.**

**Control:**


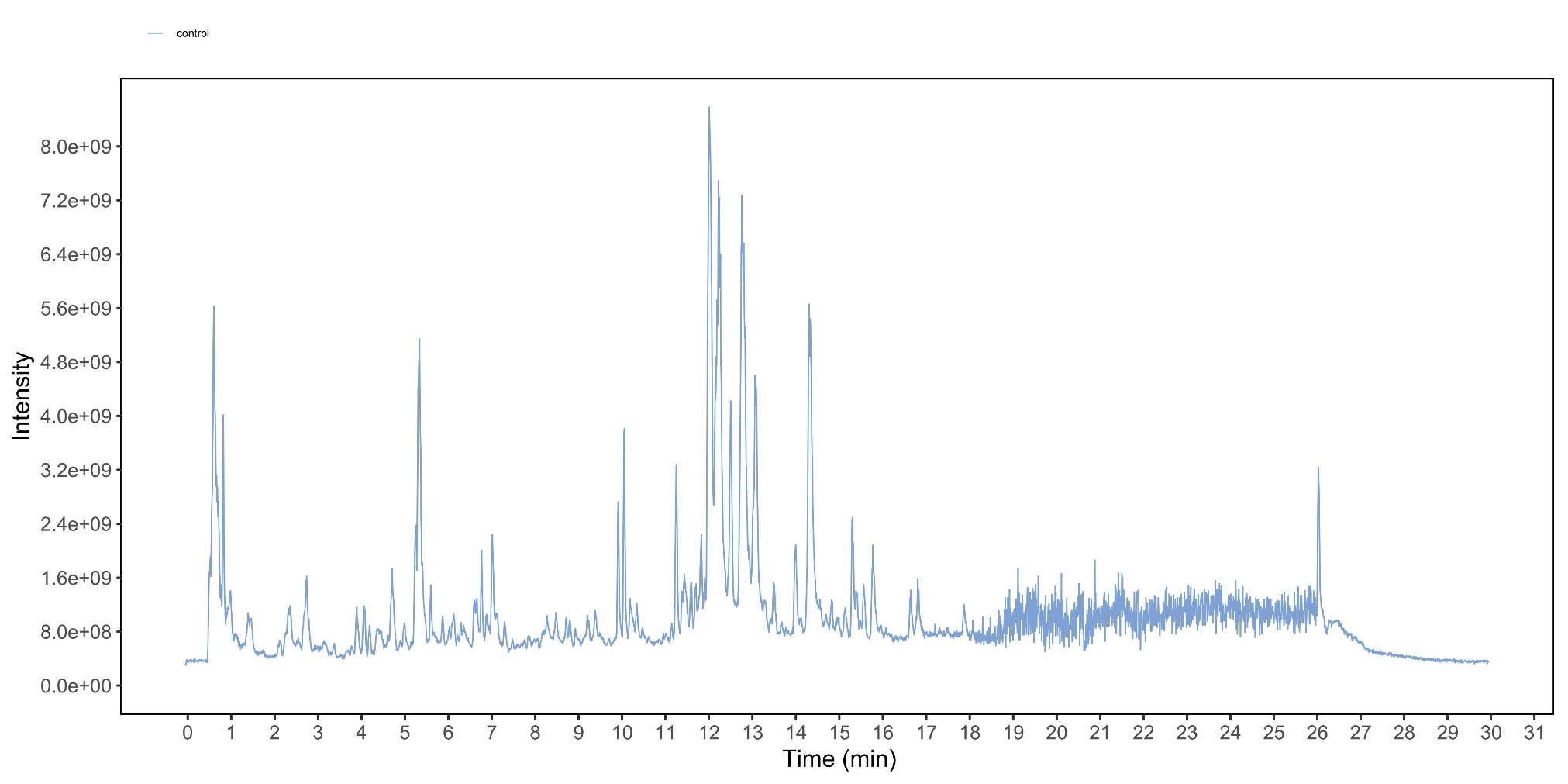


PPL extract:


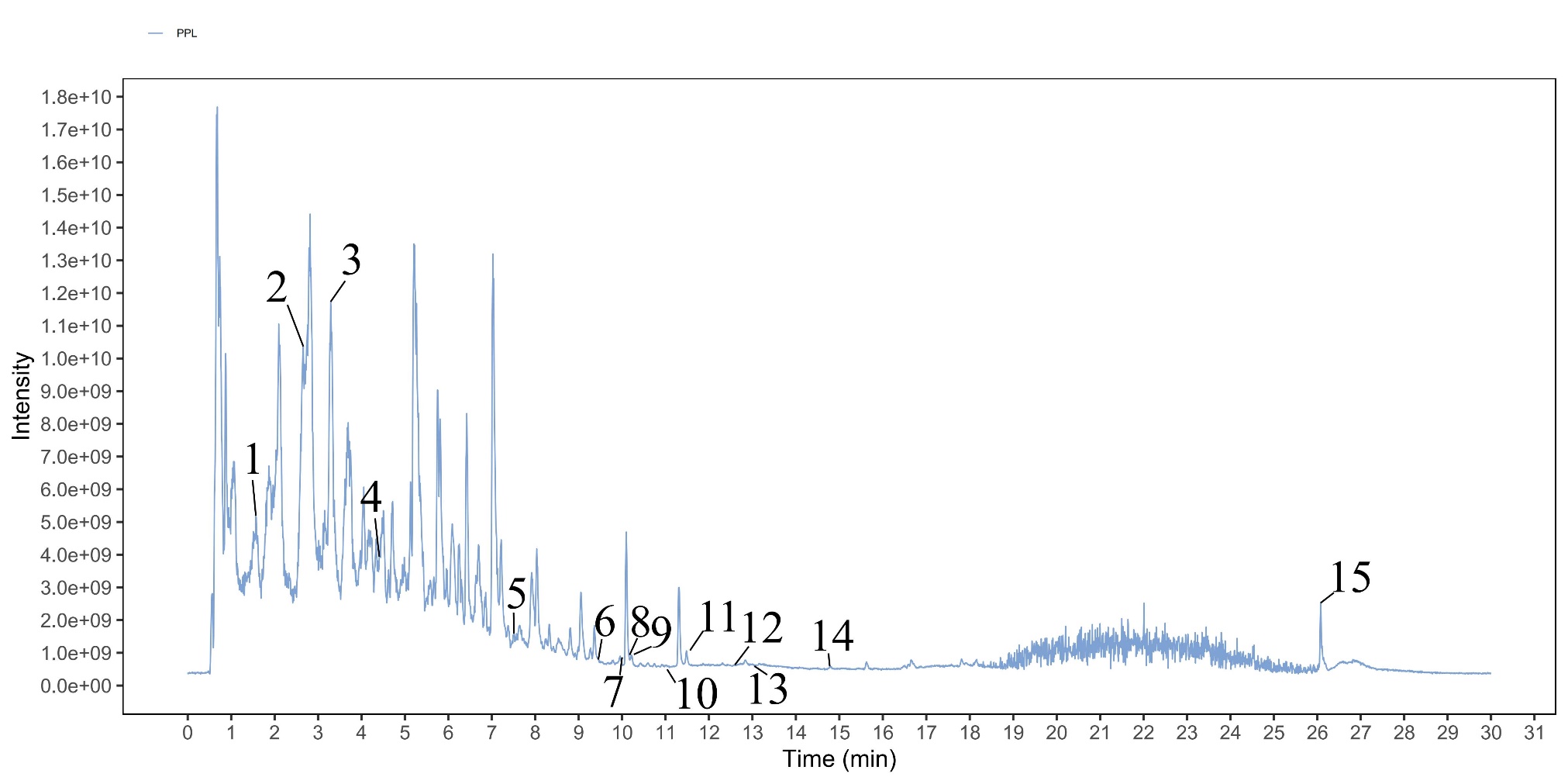


PPL metabolites：


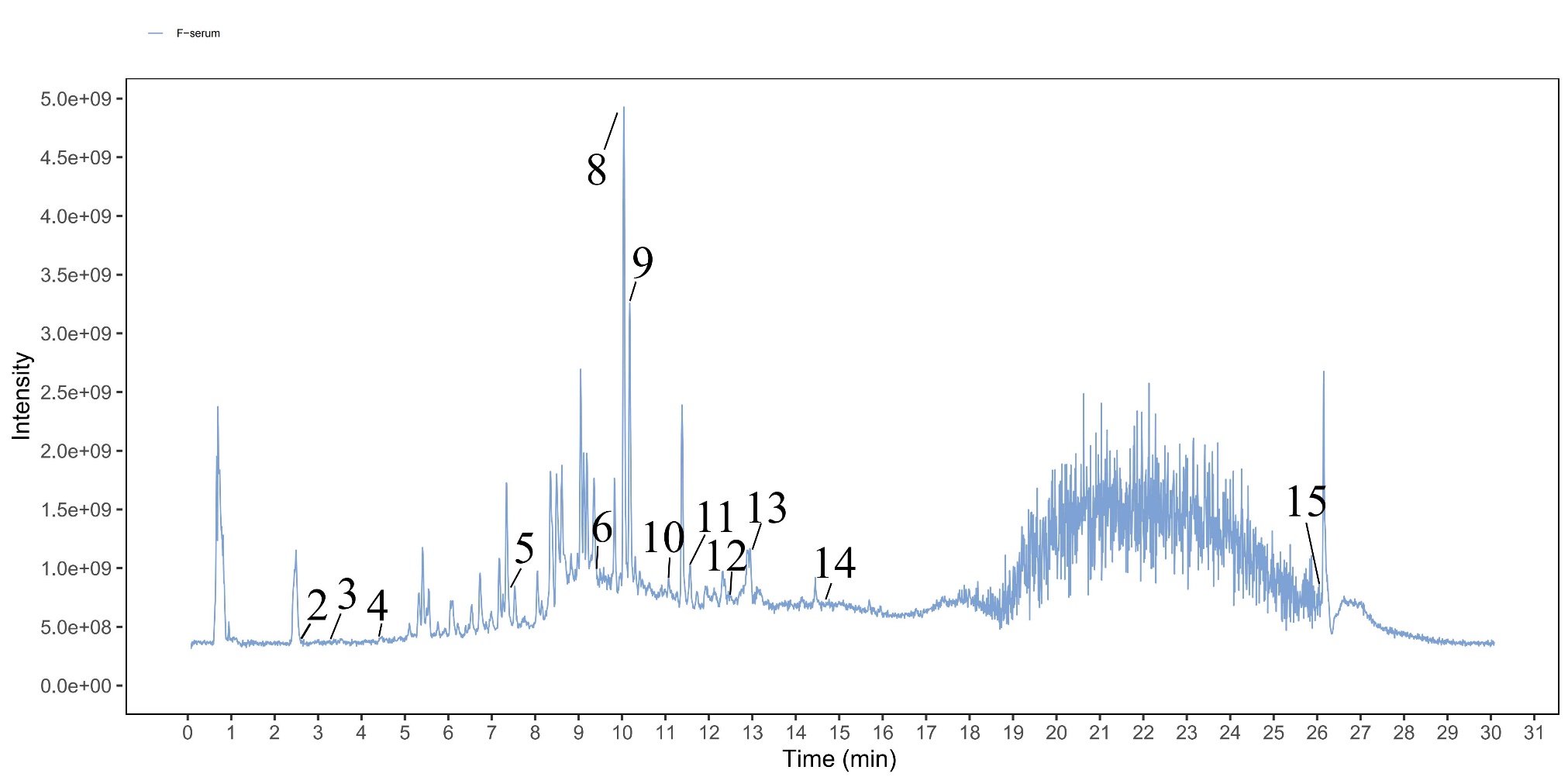


**Figure S1:**

| **1** | 2-METHYLMALEATE | **9** | Genistein |
| --- | --- | --- | --- |
| **2** | 4-Hydroxybenzoic acid | **10** | Cholic acid |
| **3** | 4-hydroxybenzaldehyde | **11** | Melibiose |
| **4** | Para-cresol | **12** | Protocatechualdehyde |
| **5** | Kaempferol | **13** | Baicalin |
| **6** | Isobutyl4-hydroxybenzoate | **14** | Ecliptasaponin A |
| **7** | Pectolinarigenin | **15** | Fumaric acid |
| **8** | Chlorogenic acid |  |  |

**Figure S2**

**UPLC/QE-HFX base peak intensity chromatograms of PPL(B) and PPL extract (C) in positive mode.**

**Control:**


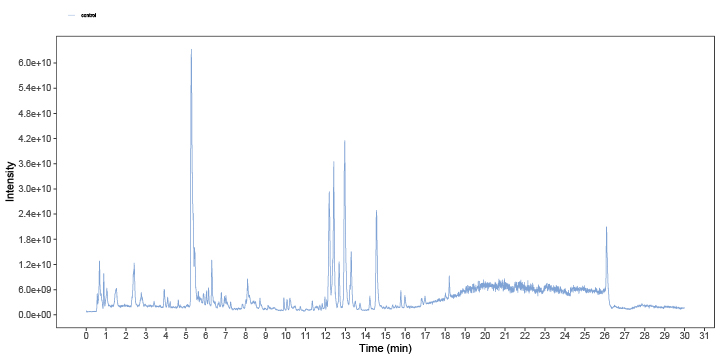


PPL extract:


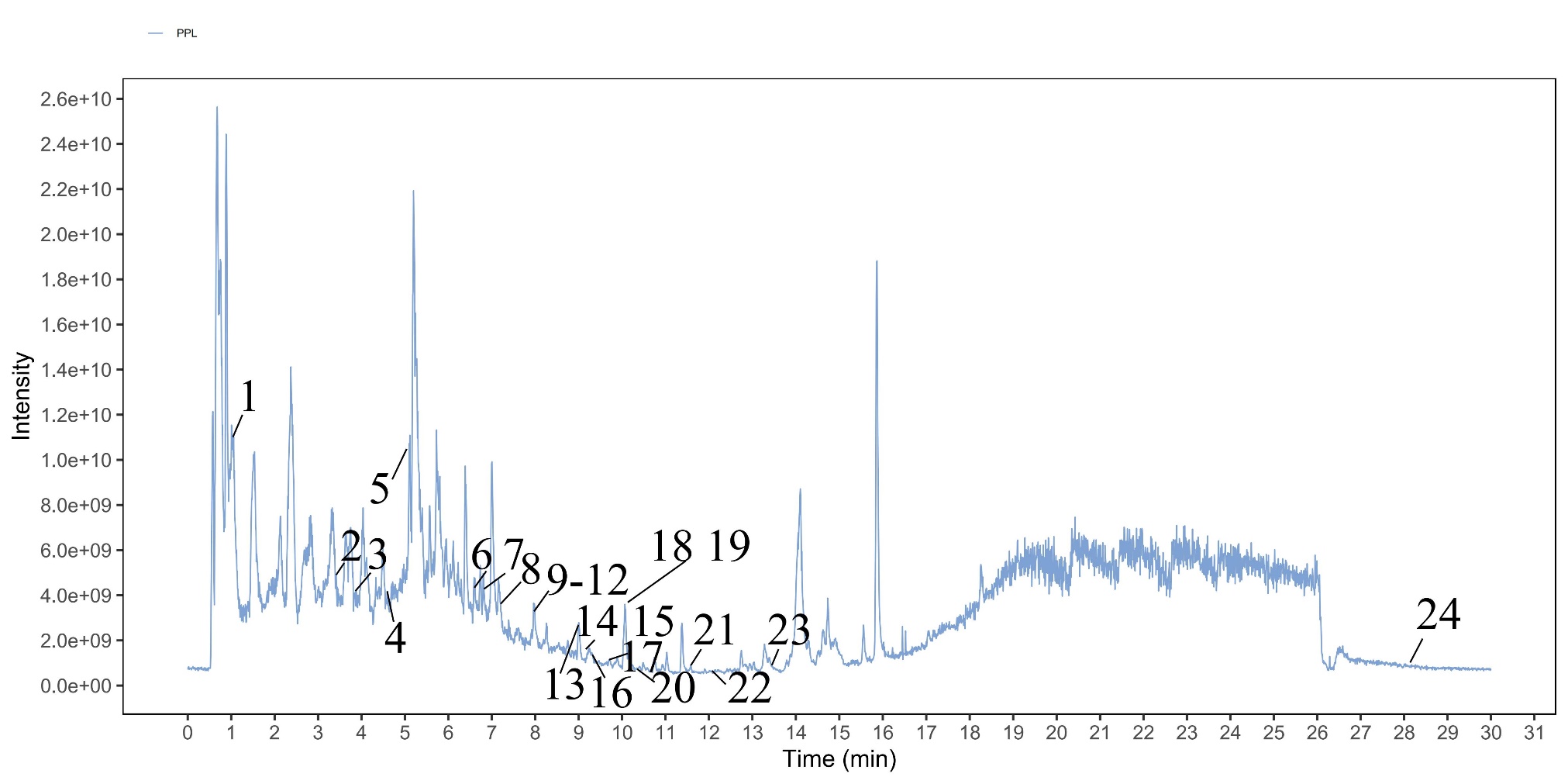


PPL metabolites:


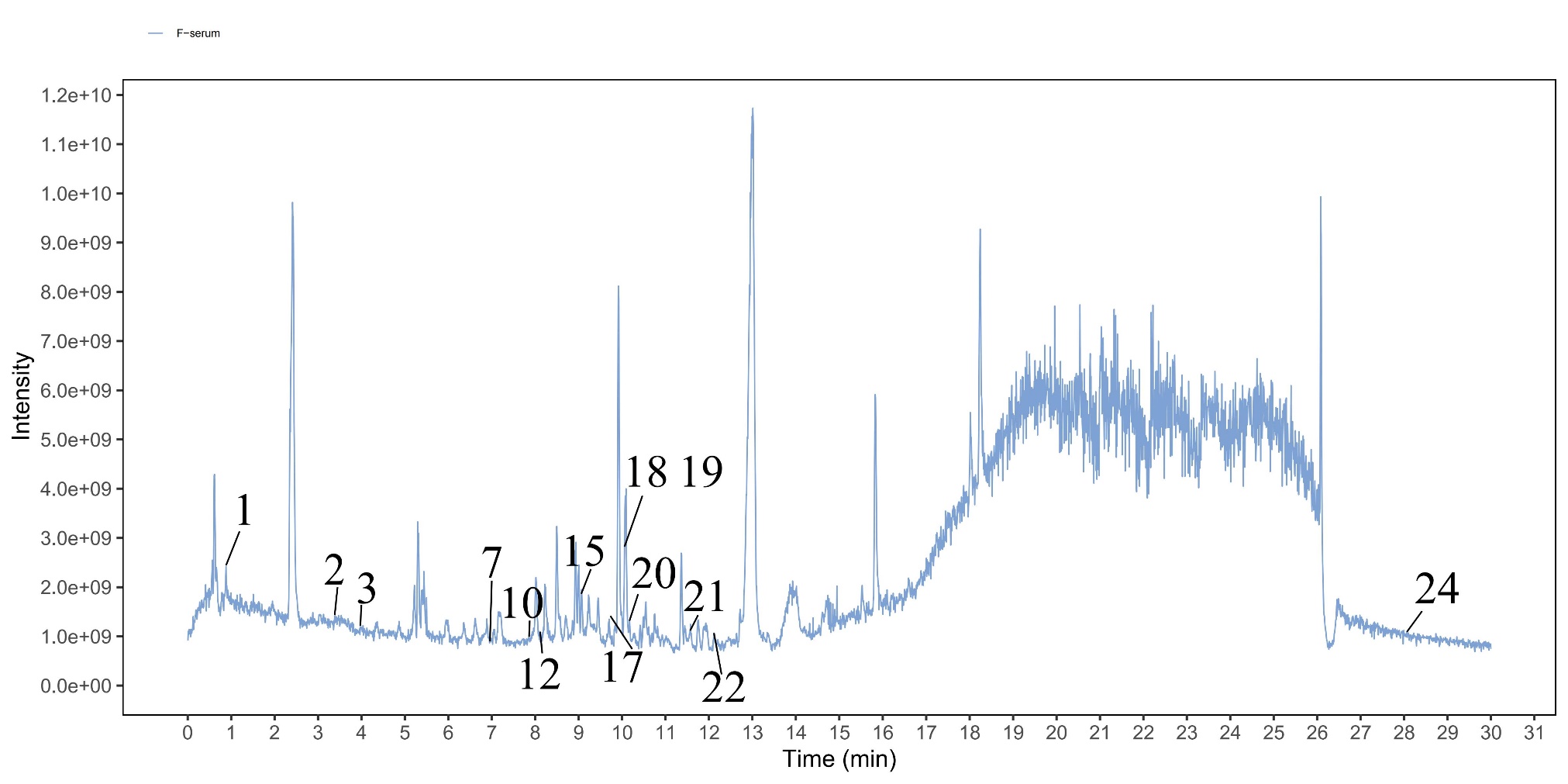


**Figure S2:**

| **1** | Guanine | **13** | M2 |
| --- | --- | --- | --- |
| **2** | picein | **14** | Quercetin |
| **3** | Isatin | **15** | Apigenin |
| **4** | Myricetin | **16** | 4-Methylesculetin |
| **5** | M1 | **17** | Galangin |
| **6** | Skimmin | **18** | Emodin |
| **7** | Hydroxygenkwanin | **19** | Baicalin |
| **8** | Demethylwedelolactone | **20** | Baicalein |
| **9** | Hydroxygenkwanin | **21** | Deoxycholic Acid |
| **10** | Abscisic acid | **22** | Apigenin 7-O-glucuronide |
| **11** | Phloretin | **23** | 3-O-Acetyldiosgenin |
| **12** | Kaempferol | **24** | Nicotinic acid |

M1:5,7-dihydroxy-2-(4-hydroxyphenyl)-3-[(2S,3R,4S,5S,6R)-3,4,5-trihydroxy-6-[[(2S,3R,4S,5S)-3,4,5-trihydroxyoxan-2-yl]oxymethyl]oxan-2-yl]oxychromen-4-one

M2: 5,7-dihydroxy-2-(4-hydroxy-3-methoxyphenyl)-6-(3-methylbut-2-enyl)-2,3-dihydrochromen-4-one

**Figure S3:**


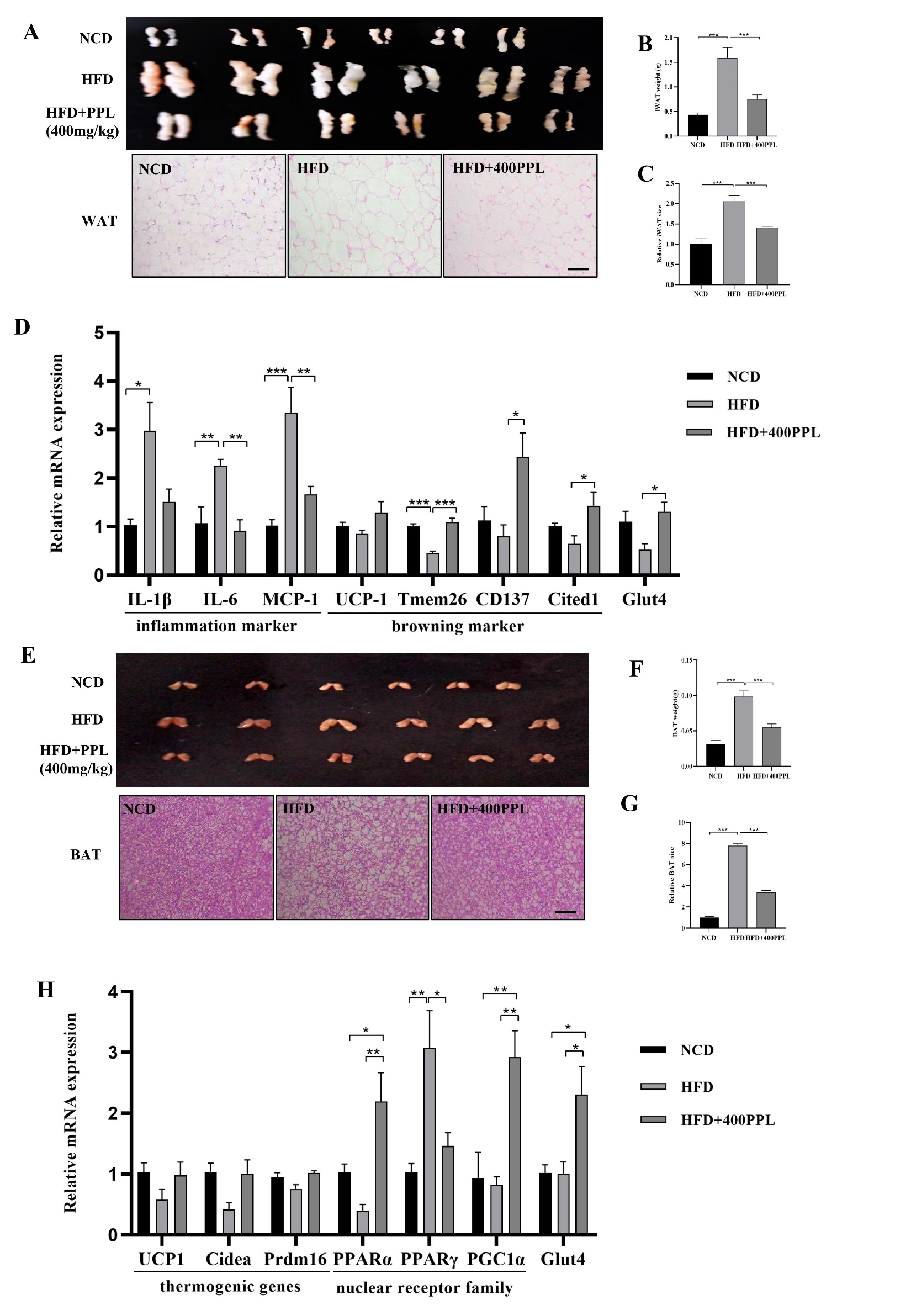


**Figure S4**

**HFD+400PPL**

**HFD**

**NCD**

**
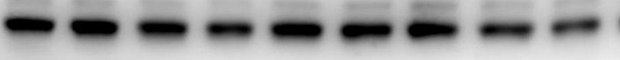

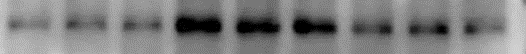

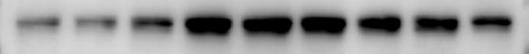

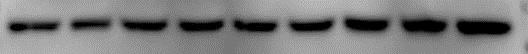
**

**98**

HMGCR

**57**

CYP7A1

**132**

SREBP2

Actin

**42**
